# Knockdown of ketohexokinase versus inhibition of its kinase activity exert divergent effects on fructose metabolism

**DOI:** 10.1172/jci.insight.184396

**Published:** 2024-12-06

**Authors:** Se-Hyung Park, Taghreed Fadhul, Lindsey R. Conroy, Harrison A Clarke, Ramon C. Sun, Kristina Wallenius, Jeremie Boucher, Gavin O’Mahony, Alessandro Boianelli, Marie Persson, Sunhee Jung, Cholsoon Jang, Analia S. Loria, Genesee J. Martinez, Zachary A. Kipp, Evelyn A. Bates, Terry D. Hinds, Senad Divanovic, Samir Softic

**Affiliations:** 1 Department of Pediatrics and Division of Pediatric Gastroenterology and; 2 Department of Molecular and Cellular Biochemistry, University of Kentucky, Lexington, Kentucky, USA.; 3 Department of Biochemistry & Molecular Biology, Center for Advanced Spatial Biomolecule Research, University of Florida, Gainesville, Florida, USA.; 4 Bioscience, Research and Early Development, Cardiovascular, Renal and Metabolism, BioPharmaceuticals R&D, AstraZeneca, Gothenburg, Sweden.; 5 The Lundberg Laboratory for Diabetes Research, Department of Molecular and Clinical Medicine, Sahlgrenska Academy, University of Gothenburg, Gothenburg, Sweden.; 6 Medicinal Chemistry and; 7 DMPK, Research and Early Development, Cardiovascular, Renal and Metabolism, BioPharmaceuticals R&D, AstraZeneca, Gothenburg, Sweden.; 8 Department of Biological Chemistry, School of Medicine; and Center for Epigenetics and Metabolism, Chao Family Comprehensive Cancer Center, University of California, Irvine, Irvine, California, USA.; 9 Department of Pharmacology and Nutritional Sciences, University of Kentucky, Lexington, Kentucky, USA.; 10 Department of Pediatrics, University of Cincinnati College of Medicine; and Division of Immunobiology, Cincinnati Children’s Hospital Medical Center, Cincinnati, Ohio, USA.; 11 Joslin Diabetes Center and Department of Medicine, Harvard Medical School, Boston, Massachusetts, USA.

**Keywords:** Hepatology, Metabolism, Carbohydrate metabolism, Hepatitis, Obesity

## Abstract

Excessive fructose intake is a risk factor for the development of obesity and its complications. Targeting ketohexokinase (KHK), the first enzyme of fructose metabolism, has been investigated for the management of metabolic dysfunction–associated steatotic liver disease (MASLD). We compared the effects of systemic, small molecule inhibitor of KHK enzymatic activity with hepatocyte-specific, N-acetylgalactosamine siRNA–mediated knockdown of KHK in mice on an HFD. We measured KHK enzymatic activity, extensively quantified glycogen accumulation, performed RNA-Seq analysis, and enumerated hepatic metabolites using mass spectrometry. Both KHK siRNA and KHK inhibitor led to an improvement in liver steatosis; however, via substantially different mechanisms, KHK knockdown decreased the de novo lipogenesis pathway, whereas the inhibitor increased the fatty acid oxidation pathway. Moreover, KHK knockdown completely prevented hepatic fructolysis and improved glucose tolerance. Conversely, the KHK inhibitor only partially reduced fructolysis, but it also targeted triokinase, mediating the third step of fructolysis. This led to the accumulation of fructose-1 phosphate, resulting in glycogen accumulation, hepatomegaly, and impaired glucose tolerance. Overexpression of wild-type, but not kinase-dead, KHK in cultured hepatocytes increased hepatocyte injury and glycogen accumulation after treatment with fructose. The differences between KHK inhibition and knockdown are, in part, explained by the kinase-dependent and -independent effects of KHK on hepatic metabolism.

## Abstract 2

The KHK inhibitor and KHK siRNA improve hepatic steatosis via different mechanisms. The inhibitor enhances the FAO pathway, while KHK siRNA lowers the DNL pathway.

## Introduction

High intake of dietary sugar has undoubtedly been linked to the development of obesity-associated complications, such as metabolic dysfunction–associated steatotic liver disease (MASLD). Therefore, the World Health Organization (1), and several leading medical societies, such as the American Heart Association (2), the Canadian Diabetes Association (3), and 3 European medical groups (EASL, EASD, EASO) (4), recommend reducing sugar intake as a way to ameliorate metabolic dysfunction.

The adverse effects of sugar have mainly been attributed to its fructose component. Several properties of dietary fructose make it uniquely suitable to promote the development of obesity-associated complications, such as MASLD (5). Fructose strongly enhances hepatic de novo lipogenesis (DNL) by acting as a substrate for fatty acid synthesis and by upregulating SREBP1c and carbohydrate response element binding protein (ChREBP) lipogenic transcription factors (6, 7). On the other hand, fructose restriction improves hepatic steatosis (8) and lowers body mass index (9). Furthermore, fructose decreases fatty acid oxidation (FAO) (10), either indirectly through DNL intermediate malonyl-CoA or directly through suppression of carnitine palmitoyltransferase 1a (CPT1α) (11). Additionally, fructose has been proposed to induce hepatic insulin resistance (12), elevate uric acid production (13), promote endoplasmic reticulum stress (14), and propagate mitochondrial dysfunction (15). Indeed, fructose consumption is 2- to 3-fold higher in adults with biopsy-confirmed MASLD (16) and correlates with a higher incidence of liver fibrosis (17).

The effects of dietary fructose are dependent on its catabolism via ketohexokinase (KHK), the first enzyme of fructolysis. Thus, mice with whole-body knockout (KO) of KHK on an obesogenic diet are protected from MASLD (18, 19). We and others have shown that liver-specific deletion of KHK is sufficient to reverse MASLD (7, 20). The strong premise that fructose contributes to metabolic dysfunction and that KHK deletion prevents it has led to the development of small molecule inhibitors of KHK enzymatic activity. The inhibitors target the ATP binding domain of KHK, which prevents fructose phosphorylation to fructose-1 phosphate (F1P) (21). Phosphorylation of fructose, similar to the phosphorylation of glucose, is required for its downstream metabolism. An early KHK inhibitor was efficacious in preclinical studies (22), but this compound has not been tested in clinical trials. Many enzymes contain an ATP binding domain, so the initial inhibitors were not selective enough for KHK activity. Within the last decade, a new class of compounds, allegedly 600 times more specific for KHK activity, have been developed. These inhibitors were likewise found to effectively reduce steatosis in rats (23) but also in human liver tissue (24). Moreover, recent clinical trials reported that the new KHK inhibitors significantly reduced whole liver fat in patients with MASLD (25, 26). Despite the positive data, the leading developer unexpectedly announced that it was stopping further advancement of the KHK inhibitor (27).

In this study, we compared head-to-head the effects of systemic, small molecule KHK inhibitor (PF-06835919) versus liver-specific knockdown (KD) of KHK using N-acetylgalactosamine–conjugated (GalNAc-conjugated) siRNA. Our laboratory was the first to suggest that KHK has kinase-independent functions (28, 29). We hypothesize that inhibiting only the kinase function of KHK may not be sufficient to fully reverse metabolic dysfunction associated with fructose intake. This hypothesis may explain why KHK inhibitors have not progressed in clinical development despite preclinical studies consistently showing the benefits of KHK KO.

## Results

### Inhibition of KHK activity, but not its KD, leads to hepatic glycogen accumulation.

**Figure 1:**
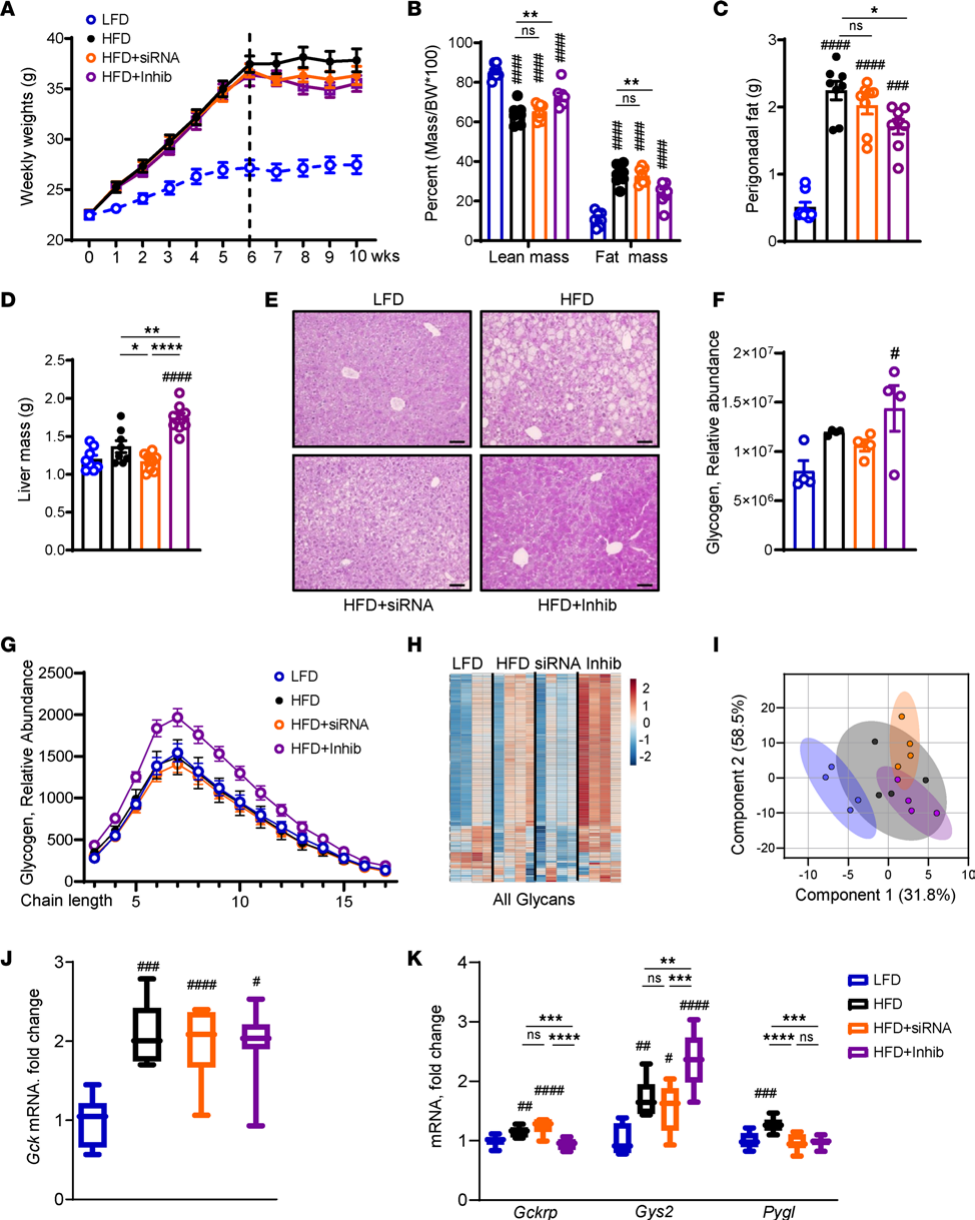
Inhibition of KHK activity, but not its KD, leads to hepatic glycogen accumulation. (**A**) Weight gain of mice on low-fat diet (LFD), high-fat diet (HFD), HFD treated with siRNA, and HFD treated with inhibitor for the last 4 weeks of this 10-week experiment. (**B**) Lean mass and fat mass normalized by body weight as assessed by EchoMRI. Perigonadal adipose tissue (**C**) and liver (**D**) weights at the time of sacrifice. *n* = 7–8 mice per group. (**E**) Representative periodic acid–Schiff (PAS) stained images of liver histology. Bar = 50 μm. (**F**) Mass spectrometry (MS) analysis for glycogen in the liver. (**G**) Glycogen chain length as determined by MS. (**H**) Heatmap of all glycans and (**I**) principal component analysis of all glycans in LFD, HFD, HFD + siRNA, and HFD + Inhib groups. *n* = 4 mice per group. mRNA expression of Gck (**J**) and the genes involved in (**K**) glycogen synthesis and degradation. *n* = 6 mice per group. Statistical analysis was performed using 1-way ANOVA compared with LFD group (^#^*P* < 0.05; ^##^*P* < 0.01; ^###^*P* < 0.001; ^####^*P* < 0.0001) with post hoc 2-tailed *t* tests between the individual groups (*P < 0.05; **P < 0.01; ***P < 0.001; ****P < 0.0001).

To compare the effects of liver-specific KHK KD using siRNA versus systemic small molecule inhibitor of KHK kinase activity, we placed male C57BL6/J mice on an LFD or an HFD for 6 weeks. Thereafter, the mice on an HFD were subdivided into 3 groups: the control group (HFD), the KHK siRNA group (HFD + siRNA), and the KHK inhibitor group (HFD + Inhib). All mice were injected with luciferase control siRNA (10 mg/kg) or siRNA targeting total KHK (20 mg/kg) every 2 weeks. Additionally, all mice were gavaged with methylcellulose control or (15 mg/kg) KHK inhibitor (PF-06835919) in methylcellulose, twice daily for 4 weeks (Supplemental Figure 1A; supplemental material available online with this article; https://doi.org/10.1172/jci.insight.184396DS1). The inhibitor dose was set to achieve exposure in mice that had previously been shown to achieve over 90% KHK inhibition in rats (23). The mice were treated with the drugs for 4 weeks. The mice in the HFD group weighed significantly more than the mice on an LFD (37.8 ± 1.1 g vs. 27.5 ± 0.9 g) (Figure 1A and Supplemental Figure 1B). KD of KHK (36.3 ± 0.9 g) or inhibition of its kinase activity (35.5 ± 0.8 g) did not significantly reduce body weight. Caloric intake was increased in all mice on an HFD, but there was no difference among the groups (Supplemental Figure 1C). Similarly, water intake was not different among the HFD groups (Supplemental Figure 1D). Body composition assessed by EchoMRI revealed a lower percentage lean mass in the HFD (66.8% ± 3.3%), compared with the LFD group (85.4% ± 1.2%) (Figure 1B). KD of KHK induced no change (65.1% ± 1.4%), while inhibition of KHK increased the percentage lean mass (72.9% ± 1.8%). Conversely, the percentage body fat was higher in mice on an HFD (30.5% ± 3.2%) compared with the LFD group (11.3% ± 1.3%). The HFD + siRNA group (32.6% ± 1.4%) was not different, while the percentage fat decreased in the HFD + Inhib group (24.2% ± 2.0%). This could be accounted for by lower perigonadal adipose tissue weight in the HFD + Inhib group (Figure 1C), while increased lean mass likely reflects higher liver weight in these mice (Figure 1D). Besides the liver, the kidneys and intestines effectively metabolize fructose via KHK (30). Kidney weight was not altered (Supplemental Figure 1E), and intestinal histology was unchanged among the groups (Supplemental Figure 1F).

A surprising increase in liver weight in the HFD + Inhib group could not be explained by steatosis (Supplemental Figure 1G), as Oil Red O staining of lipids (Supplemental Figure 1H) and liver triglycerides (Supplemental Figure 1I) decreased in both the HFD + siRNA and HFD + Inhib groups, compared with the HFD. In agreement with liver steatosis, serum triglycerides decreased in both the KHK siRNA and inhibitor groups (Supplemental Figure 2A), while serum cholesterol, which in mice mainly consists of HDL-cholesterol, was increased only in the inhibitor-treated mice (Supplemental Figure 2B). The increased liver weight agreed with the periodic acid–Schiff (PAS) staining, suggesting that glycogen was higher in the HFD + Inhib group (Figure 1E). Indeed, mass spectrometry (MS) quantification revealed elevated glycogen levels only in the HFD + Inhib group, compared with the LFD group (Figure 1F). Glycogen accumulation is partially controlled by glucagon. We found reduced glucagon concentration in the HFD + Inhib group (39 ± 4 pg/mL), compared with both HFD (63 ± 7 pg/mL) and HFD + siRNA groups (62 ± 6 pg/mL) (Supplemental Figure 2C). Next, we quantified the glycogen chain length and found that only the HFD + Inhib group had an increased abundance of glycogen composed of short glucose polymers (Figure 1G). N-linked glycans are homo- or heteropolymers of monosaccharides attached to a protein. A heatmap showed glycans were higher in the HFD compared with the LFD group (Figure 1H). Glycans decreased in the HFD + siRNA group but were the highest in the HFD + Inhib group, indicative of greater monosaccharide availability. Principal component analysis of all glycans verified differential clustering in the HFD versus LFD groups and with KHK inhibition versus KD (Figure 1I). Together, these data indicate that mice in the HFD + Inhib group had increased glycogen accumulation and greater monosaccharide availability.

To interrogate the mechanism behind increased glycogen accumulation, we quantified the expression of glucokinase (Gck), which was increased 2-fold in all mice on HFD but was not altered by KHK KD or inhibition (Figure 1J). In its inactive state, Gck is sequestered in the nucleus by glucokinase regulatory protein (Gckrp). The HFD and HFD + siRNA groups had 1.3- to 1.4-fold higher Gckrp expression than the LFD group (Figure 1K). The HFD + Inhib group did not have elevated Gckrp mRNA, suggesting that GCK was more active. Indeed, Gck activity was elevated in the HFD + Inhib group (Supplemental Figure 2D). Gck activity stimulates glycogen synthase (Gys2), which was 1.5- to 1.7-fold higher in the HFD and HFD + siRNA groups and 2.4-fold higher in the HFD + Inhib group (Figure 1K). Conversely, glycogen phosphorylase (Pygl), which liberates glucose from glycogen, was only elevated in mice on an HFD. In summary, both KD and inhibition of KHK improved hepatic steatosis, but KHK inhibition increased liver weight, in part due to higher glycogen accumulation.

Similar effects were also observed in mice fed a high-fat, high-sugar diet. The mice consumed 60% HFD supplemented with 30% fructose in drinking water for 10 weeks and were treated with KHK siRNA or the inhibitor for the last 4 weeks, as before. Both KHK siRNA and the inhibitor treatment reduced body weight (Supplemental Figure 2E). This was accompanied by lower liver weight in the siRNA-treated but not in the inhibitor-treated group (Supplemental Figure 2F). In fact, when normalized to body weight, the liver mass increased in the inhibitor group (Supplemental Figure 2G), as it did in mice on HFD. There was no difference in the weight of epididymal adipose tissue (Supplemental Figure 2H), while subcutaneous adipose tissue was lower in the inhibitor-treated mice (Supplemental Figure 2I).

### Small molecule inhibitor decreases KHK enzymatic activity in the liver and kidney but not in the intestine.

**Figure 2:**
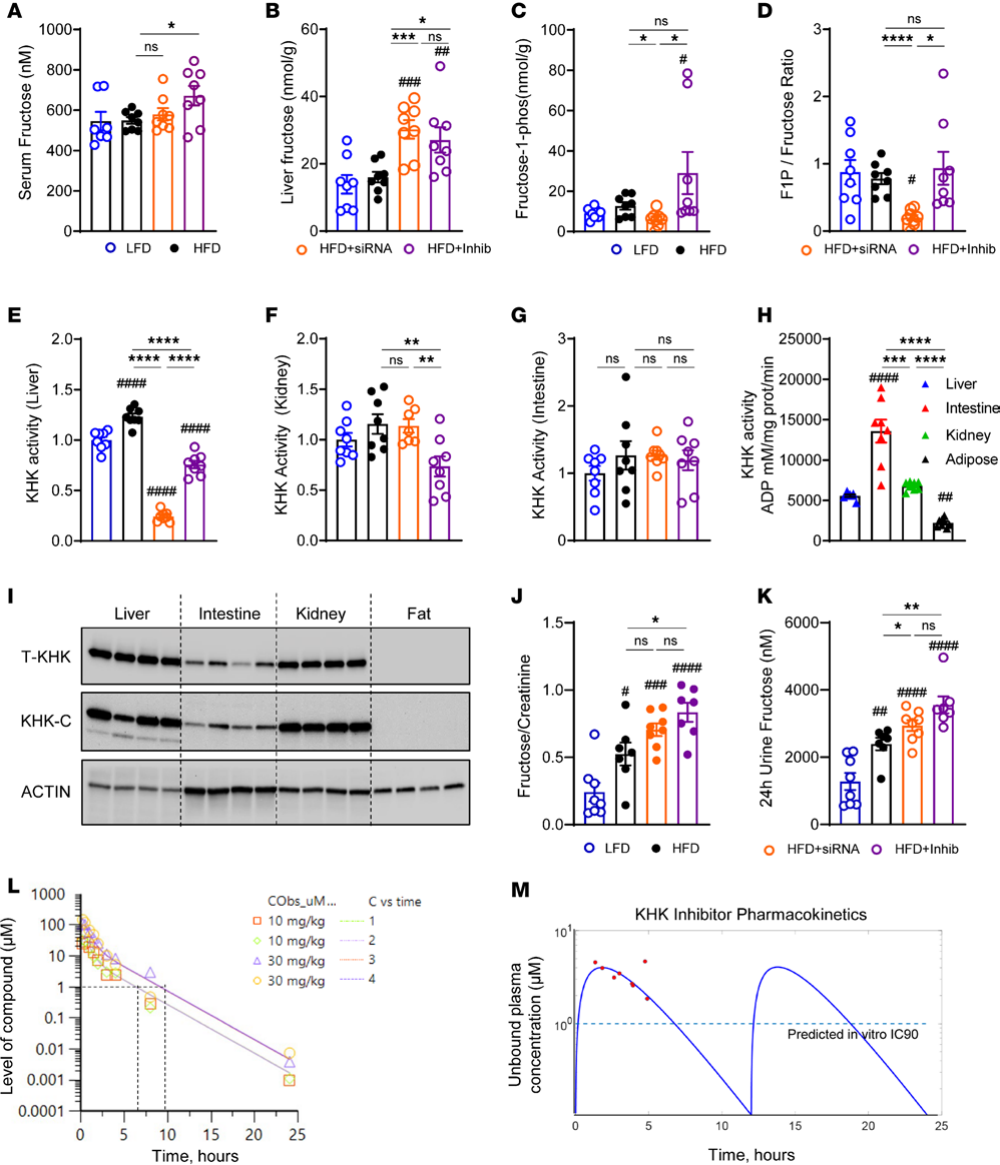
Small molecule inhibitor decreases KHK enzymatic activity in liver and kidney but not in intestine. (**A**) Serum fructose level from the mice at sacrifice. The levels of (**B**) fructose (**C**) and fructose 1-phosphate (F1P) and the ratio of (**D**) F1P/fructose in the liver. *n* = 7–8 mice per group. Quantification of KHK activity in (**E**) liver, (**F**) kidney, and (**G**) intestine. (**H**) Absolute KHK activity in liver, intestine, kidney, and perigonadal adipose tissue. (**I**) Western blot of total KHK and KHK-C in liver, intestine, kidney, and perigonadal adipose tissue. *n* = 4 mice per group. Actin was used as a loading control. (**J**) Urinary fructose level corrected by urine creatinine and (**K**) fructose excretion in urine over 24 hours. (**L**) In vivo monitoring of the inhibitor concentration over 24 hours following single gavage with 10 mg/kg or 30 mg/mL of the inhibitor. *n* = 2 mice per group. (**M**) Unbound plasma concentration of the inhibitor (red dots) quantified by MS in LFD-fed mice, 2–5 hours after last dose of the inhibitor. Pharmacokinetic model fitting (blue line) based on inhibitor concentration (red dots). Dashed line represents target concentration. Statistical analysis was performed using 1-way ANOVA compared with the LFD group (^#^*P* < 0.05; ^##^*P* < 0.01; ^###^*P* < 0.001; ^####^*P* < 0.0001) with post hoc 2-tailed *t* tests between the individual groups (*P < 0.05; **P < 0.01; ***P < 0.001; ****P < 0.0001).

Due to the phenotypic differences between KHK KD and inhibition of its kinase activity, we assessed how well these modalities prevent hepatic fructose metabolism. Compared with the HFD-fed mice (549 ± 17 nM), serum fructose was elevated in HFD + Inhib mice (672 ± 48 nM) (Figure 2A), in line with systemic inhibition of fructose metabolism. Liver-specific KHK KD (580 ± 31 nM) did not increase serum fructose, as fructose can also be metabolized in other tissues. As expected, liver fructose concentration was higher in both HFD + siRNA (30.0 ± 2.8 nmol/g) and HFD + Inhib (27.1 ± 3.8 nmol/g) groups, compared with the HFD group (16.1 ± 1.6 nmol/g) (Figure 2B). The product of KHK activity, F1P, was reduced in the HFD + siRNA group (6.2 ± 1.3 nmol/g), compared with the HFD group (12.7 ± 1.8 nmol/g) (Figure 2C). Surprisingly, the HFD + Inhib group (29.0 ± 10.4 nmol/g) had a much higher F1P concentration 2–5 hours after the inhibitor gavage. F1P is known to induce liver injury (31), so we quantified serum alanine aminotransferase (ALT). The HFD group had higher ALT than the LFD group, but ALT decreased in both HFD + siRNA and HFD + Inhib groups (Supplemental Figure 3A). The expression of proinflammatory genes was also increased in the HFD, compared with the LFD, group but was largely unaffected by the 2 interventions, except monocyte chemoattractant protein–1, which decreased in both HFD + siRNA and HFD + Inhib groups (Supplemental Figure 3B). The expression of profibrotic genes collagen type I alpha 1 chain and α–smooth muscle actin (α-Sma) was unaffected, while TGF-β increased in mice on HFD and decreased with both interventions (Supplemental Figure 3C).

Next, we calculated the F1P-to-fructose ratio to approximate KHK activity. The ratio was reduced only in the KHK + siRNA group, but it was unchanged in the HFD + Inhib group (Figure 2D). Hepatic KHK activity was also directly measured utilizing our recently developed protocol (32). The mice on an HFD had 23% higher KHK activity in the liver, compared with the mice on an LFD (Figure 2E). Compared with the HFD group, KD of KHK decreased KHK activity by 80%, likely reaching the background levels of the assay. However, the inhibitor lowered KHK activity by only 38%. These data agree with the F1P/fructose ratio, verifying that the inhibitor group can still metabolize some fructose in the liver. In the kidney, only the inhibitor, but not siRNA, decreased KHK activity by 36% (Figure 2F). However, in the intestine, neither intervention lowered KHK activity (Figure 2G). We compared KHK activity in the liver, intestine, and kidney with adipose tissue, which does not express KHK. In LFD-fed mice, KHK activity was 2.4-fold higher in the intestine, as compared with both the liver and kidneys (Figure 2H). Next, we measured the protein levels of total KHK and KHK-C isoform. The intestine had 3 times lower KHK-C protein compared with the liver and kidney, whereas adipose tissue did not express KHK (Figure 2I and Supplemental Figure 4A). These data agree with a study documenting that the intestine metabolizes fructose more robustly than the liver but can only metabolize a small amount of fructose (33). Urine fructose normalized to creatinine was 2-fold higher in the HFD compared with the LFD group (Figure 2J). The HFD + siRNA group did not show a further elevation; however, the HFD + Inhib group had a stepwise higher urinary fructose. Urine creatinine (Supplemental Figure 4B) and urine volume collected over 24 hours (Supplemental Figure 4C) were not different among the groups. Urine fructose excretion over 24 hours was higher in mice on an HFD, and it further increased following both KHK KD or inhibition of its activity (Figure 2K). In summary, the KD of KHK resulted in an almost complete loss of fructose metabolism in the liver. In contrast, systemic inhibition of KHK activity partially reduced fructose metabolism in the liver and kidney but not in the intestine.

Due to differences in KHK activity and the F1P/fructose ratio, we tested whether the dose of the inhibitor was adequate to inhibit KHK activity in vivo. In a pilot study, mice were gavaged 10 mg/kg, 30 mg/kg, or 60 mg/kg of the KHK inhibitor or methylcellulose vehicle 1 hour before a fructose challenge test using 6 mg/kg of fructose, and blood samples were collected over time. Mice gavaged with fructose and methylcellulose had elevated serum fructose excursion over 60 minutes (Supplemental Figure 4D). All 3 concentrations of the inhibitor induced a dose-dependent increase in plasma fructose levels (Supplemental Figure 4D). Serum glucose (Supplemental Figure 4E) and insulin (Supplemental Figure 4F) were the highest in mice treated with fructose and vehicle, and they decreased with the administration of 10 or 30 mg/kg of the inhibitor. Next, the level of the inhibitor in the blood was quantified over a 24-hour period in mice gavaged with 10 or 30 mg/kg of the inhibitor. Based on in vitro potency, 1 μM inhibitor concentration is required to inhibit KHK activity by 90% (IC_90_). A dose of 10 or 30 mg/kg inhibitor achieved in vivo inhibitor concentration above the IC_90_ for 7–10 hours (Figure 2L). Based on these results, the mice in our experiment were given 15 mg/kg inhibitor twice daily.

The exposure to the inhibitor was measured by MS in our experimental mice at the time of sacrifice. The pharmacokinetic model fitting profile (Figure 2M) shows the free maximum exposure of the inhibitor was 4.6 μM and the free average exposure was 1.6 μM, in line with the in vitro IC_90_ exposure Gutierrez reported (23). Despite achieving the targeted plasma concentration, the inhibitor lowered KHK activity only by 38% in our study using mice, while it decreased by 90% in the Pfizer study using rats. We found that KHK protein is 4-fold more abundant in the livers of rats compared with mice (Supplemental Figure 4, G and H). On the other hand, KHK activity per microgram of KHK was significantly higher in mice compared with rats (Supplemental Figure 4I). The higher KHK activity in mice may explain why the inhibitor did not effectively reduce KHK function in mice, as observed in rats.

### KHK siRNA specifically deletes KHK-C and increases hexokinase 2, while the inhibitor partially decreases both KHK-C and triokinase and FMN cyclase (TKFC) proteins.

**Figure 3:**
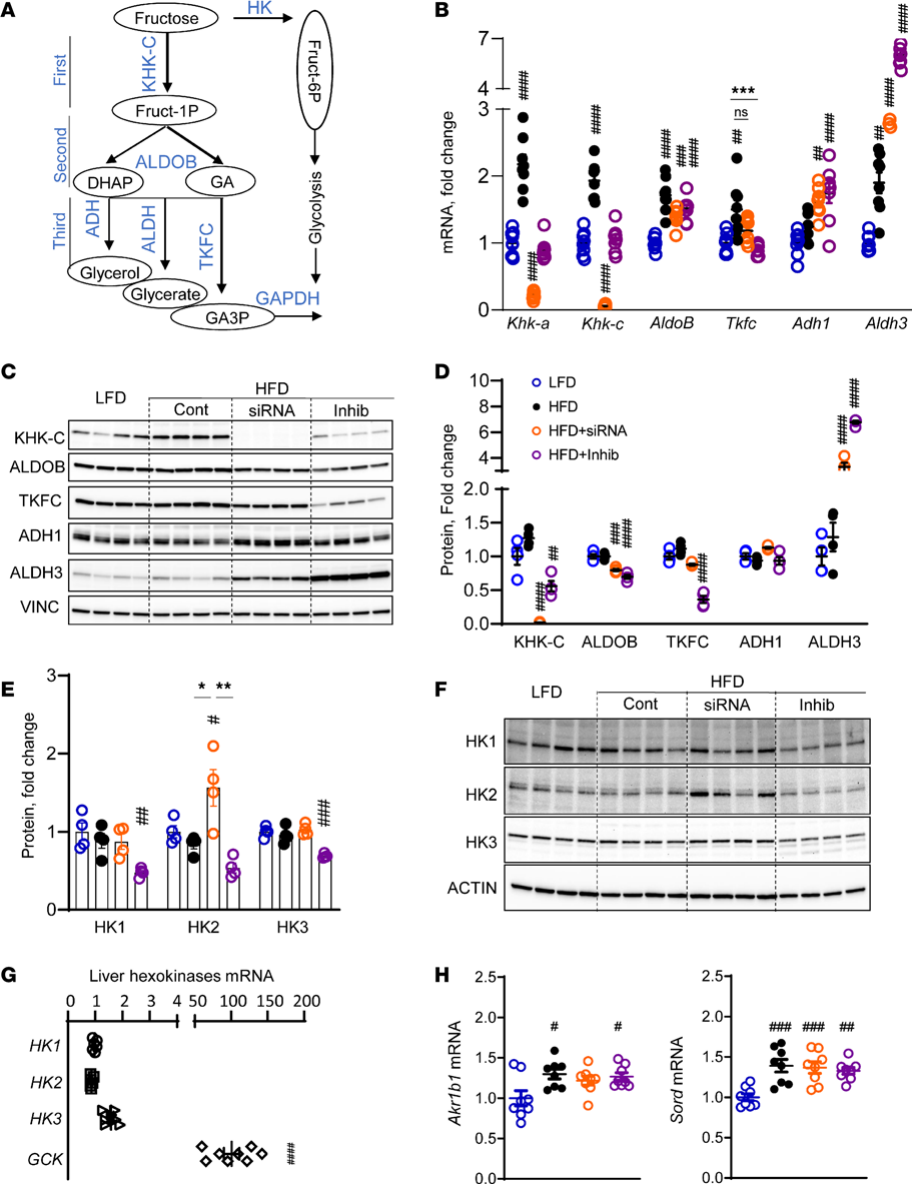
KHK siRNA completely deletes KHK-C and increases HK2, while the inhibitor partially decreases both KHK-C and TKFC proteins. (**A**) The fructose metabolism pathway. HK, hexokinase; ALDOB, aldolase B; DHAP, dihydroxyacetone phosphate; GA, glyceraldehyde; ADH, alcohol dehydrogenase; ALDH, aldehyde dehydrogenase; GA3P, glyceraldehyde 3-phosphate. (**B**) mRNA of fructose-metabolizing enzymes from livers of the mice. *n* = 6 mice per group. Tkfc, triokinase and FMN cyclase. (**C**) Western blot and (**D**) densitometry quantification of fructose metabolizing enzymes in liver lysates. *n* = 4 mice per group. (**E**) Densitometry quantification and (**F**) Western blot of HKs. *n* = 4 mice per group. (**G**) mRNA expression of HKs in the liver from mice fed an LFD. (**H**) Hepatic mRNA expression of aldo-keto reductase (Akr1b1) and sorbitol dehydrogenase (Sord). Statistical analysis was performed using 1-way ANOVA compared with the LFD group (^#^*P* < 0.05; ^##^*P* < 0.01; ^###^*P* < 0.001; ^####^*P* < 0.0001) with post hoc 2-tailed *t* tests between the individual groups (*P < 0.05; **P < 0.01; ***P < 0.001).

Given the differences in target engagement, we next interrogated the fructolysis pathway (Figure 3A). The expression of enzymes catalyzing the first, second, and third steps of fructose metabolism was increased 1.3- to 2.2-fold in mice on an HFD compared with LFD (Figure 3B). KD of KHK decreased both KHK-A and KHK-C mRNA over 90% compared with the HFD group. Interestingly, inhibition of KHK activity also decreased KHK-A and KHK-C mRNA by 45% to 58% compared with the HFD group. KHK KD or inhibition of its activity did not affect AldoB expression. The expression of the enzymes mediating the third step of fructolysis was not affected by KHK KD, except Aldh3a2, which was 63% higher compared with the HFD group. Conversely, inhibition of KHK activity decreased Tkfc expression by 40%, and it increased Adh1 by 1.4-fold and Aldh3a2 by 3.2-fold, compared with the HFD group. Similar to mRNA, KHK-C protein was increased in mice on an HFD compared with an LFD (Figure 3, C and D). The KHK + siRNA group completely lacked KHK-C protein, whereas, interestingly, the KHK + Inhib group had 56% lower KHK-C protein. ALDOB was slightly lower in both KHK + siRNA and KHK + Inhib groups. TKFC, which catalyzes a major arm of the third step of fructose metabolism, was significantly lower (*P* < 0.001) in the KHK + Inhib but not in the KHK + siRNA group. ADH1 was not different, whereas ALDH3 increased in both KHK + siRNA and KHK + Inhib groups. An unexpected decrease in TKFC protein led us to suspect that the KHK inhibitor also targets TKFC. Indeed, the KHK inhibitor was able to lower the enzymatic activity of recombinant mouse TKFC protein in a dose-dependent fashion (Supplemental Figure 5A). To show that TKFC is inhibited in vivo, we quantified hepatic GA, a substrate for TKFC (Supplemental Figure 5B). GA levels were not affected (Supplemental Figure 5C), but its metabolite, glycerate (Supplemental Figure 5D), as well as 3-phosphoglycerate (Supplemental Figure 5C) were upregulated. This agrees with elevated ALDH in the KHK + Inhib group and provides indirect evidence that TKFC is inhibited in vivo. Together, these data indicate that KHK siRNA profoundly decreased KHK-C mRNA and protein, while KHK inhibitor decreased protein and enzymatic activity of both KHK-C and TKFC.

In the absence of KHK, fructose may be metabolized by an alternative pathway where HK phosphorylates fructose to fructose-6 phosphate (F6P). There are 4 HKs, 1, 2, 3, and 4, aka Gck. The HFD + siRNA group showed an increase in HK2 mRNA (Supplemental Figure 5B) and protein levels (Figure 3, E and F), consistent with an alternate route of fructose metabolism. On the other hand, HK1, HK2, and HK3 were lower in the HFD + Inhib mice since they had partially preserved KHK activity (Figure 2E), and GCK activity was elevated in this group (Supplemental Figure 2D). The expression of HKs was much lower than Gck in the liver (Figure 3G). However, fasted fructose concentration (0.01 mM) (34) was also much lower than glucose (5 mM), so low abundance of HK2 may still play a physiologic role in the HFD + siRNA mice. An increase in all 3 steps of fructose metabolism in mice on an HFD can be explained, in part, by endogenous fructose production mediated by aldo-keto reductase (Akr1b1) and sorbitol dehydrogenase (Sord), which were increased in all mice on an HFD (Figure 3H). In summary, KHK KD more completely abolished KHK activity, leading to alternative metabolism via HK2. Conversely, the KHK inhibitor partially lowered both KHK-C and TKFC activities, which may account for increased F1P in this group.

### KD of KHK, but not inhibition of its kinase activity, improves glucose tolerance.

**Figure 4:**
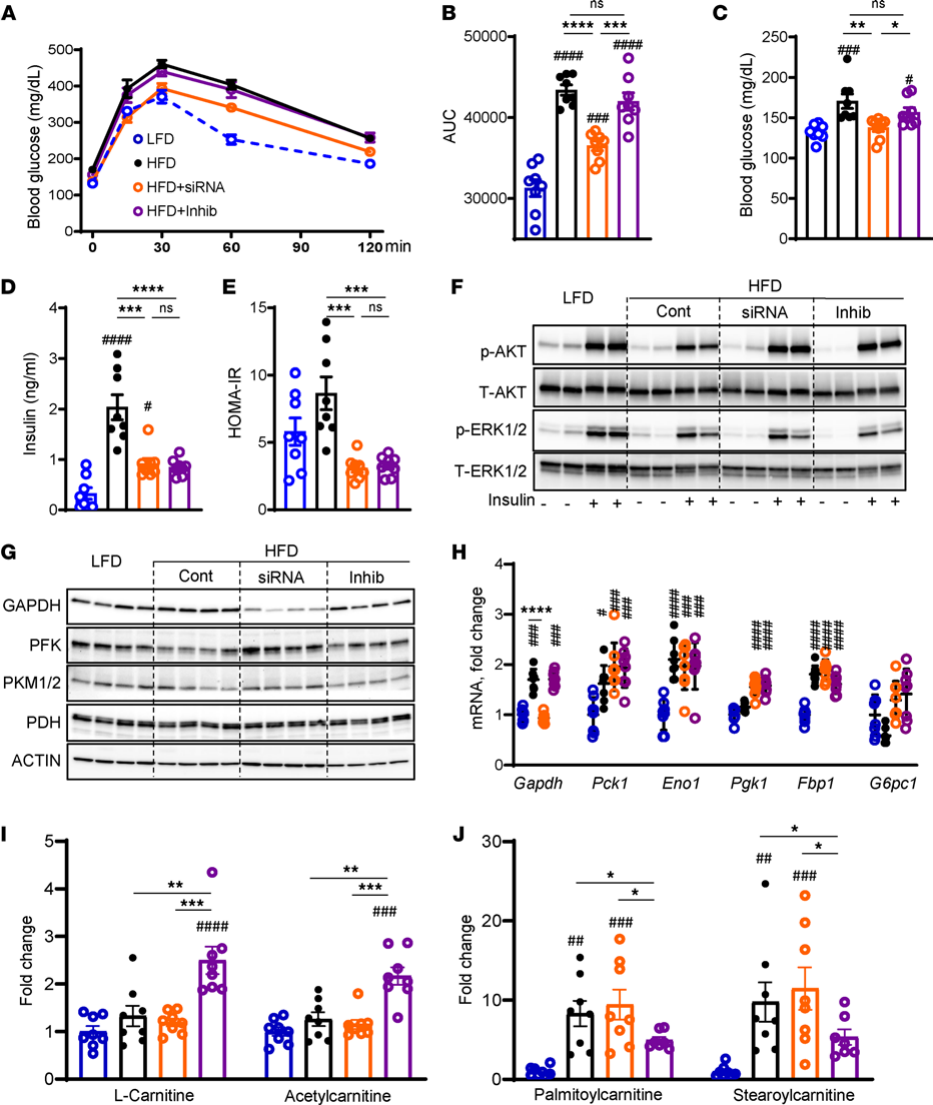
KD of KHK, but not inhibition of its activity, improves glucose tolerance and increases the glycolysis pathway. (**A**) Glucose tolerance test (GTT) measured after 8 weeks on the diets and 2 weeks after initiation of the treatments. (**B**) Area under the curve calculated from GTT. *n* = 7–8 mice per group. Fasted serum glucose (**C**), insulin (**D**), and calculated HOMA-IR (**E**) at 10 weeks on the diet. (**F**) Western blot analysis of insulin signaling in the liver. *n* = 4 mice per group. Western blot analysis (**G**) and quantitative PCR (qPCR) quantification (**H**) of genes mediating the gluconeogenesis pathway. *n* = 6 mice per group for gene expression. Actin was used as a loading control. (**I**) Short-chain and (**J**) long-chain acylcarnitines quantified by MS. *n* = 7 – 8 mice per group. Statistical analysis was performed using 1-way ANOVA compared with the LFD group (^#^*P* < 0.05; ^##^*P* < 0.01; ^###^*P* < 0.001; ^####^*P* < 0.0001) with post hoc 2-tailed *t* tests between the individual groups (*P < 0.05; **P < 0.01; ***P < 0.001; ****P < 0.0001).

The mice on an HFD had impaired glucose tolerance compared with the LFD-fed mice (Figure 4, A and B). KHK KD improved glucose tolerance, but inhibition of KHK kinase activity did not. Similarly, fasted blood glucose was elevated in mice on an HFD (171 ± 9 mg/dL) compared with the LFD group (132 ± 3 mg/dL) (Figure 4C). Glucose levels improved in the KHK siRNA (138 ± 5 mg/dL) but not the inhibitor group (157 ± 6 mg/dL). Fasted insulin was higher in the HFD (2.0 ± 0.2 ng/mL) compared with the LFD group (0.3 ± 0.1 ng/mL) and was reduced after both KHK KD (0.9 ± 0.1 ng/mL) and inhibitor treatment (0.8 ± 0.1 ng/mL) (Figure 4D). Similarly, Homeostatic Model Assessment of Insulin Resistance (HOMA-IR), a measure of whole-body insulin resistance, was elevated in mice on an HFD and was decreased in both KD and inhibitor arms (Figure 4E). Insulin-stimulated Akt phosphorylation was lower in mice on HFD compared with the LFD group (Figure 4F and Supplemental Figure 6A). KD of KHK or inhibition of its activity improved Akt phosphorylation. Erk phosphorylation was also decreased in the HFD compared with the LFD group, but it did not improve with KD or inhibition of KHK. Thus, improved glucose tolerance following KHK KD is not due to improved hepatic insulin signaling, which was equally better after both KHK KD and inhibition.

GAPDH controls the flow of fructose carbons onto the glycolysis pathway. GAPDH was slightly increased in the HFD- compared with the LFD-fed mice (Figure 4G and Supplemental Figure 6B). KHK KD profoundly lowered GAPDH, in agreement with the abrogation of fructose metabolism. Inhibition of KHK activity did not lower GAPDH. The rate-limiting enzyme of glycolysis, phosphofructokinase (PFK), was decreased in the HFD group, compared with the LFD group (Figure 4G and Supplemental Figure 6B). The mice in the HFD + siKHK group, but not in the HFD + Inhib group, had elevated PFK protein. The last enzyme of glycolysis, pyruvate kinase (PK), is allosterically activated by fructose-1,6-bisphosphate, the product of PFK activity. Therefore, PK was elevated in the HFD + siRNA group, compared with the HFD + Inhib group. Pyruvate dehydrogenase (PDH) converts pyruvate into acetyl-CoA. PDH was unchanged among the groups. Next, we assessed the gluconeogenesis pathway by qPCR. GAPDH mediates both the fructolysis and gluconeogenesis pathways. GAPDH mRNA mimicked its protein, which likely reflects its role in fructolysis (Figure 4H). All other enzymes of gluconeogenesis, phosphoenolpyruvate carboxykinase 1 (Pck1), enolase 1 (Eno1), phosphoglycerate kinase 1 (Pgk1), fructose-bisphosphatase 1 (Fbp1), and glucose-6-phosphatase (G6pc1), were not different between the HFD + siKHK and HFD + Inhib groups. Together, these data demonstrate that glucose handling is differentially regulated by KHK KD or inhibition of its kinase activity.

To further investigate the differences in glucose handling, we performed MS analysis and quantified 880 hepatic metabolites. KHK KD or inhibition of its kinase activity profoundly altered hepatic metabolome on principal component analysis (Supplemental Figure 7A). A heatmap representation of the top 110 metabolites showed that the HFD + Inhib group had an increase in acetyl-CoA and coenzyme A, substrates for DNL (Supplemental Figure 7B). On the other hand, the HFD + siRNA group had an increase in mono- and polyunsaturated fatty acids, such as 9-decenoic acid and adrenic acid (Supplemental Figure 7B). Given that the most abundant changes occurred in hepatic lipids, next, we focused on metabolites in the FAO pathway. The HFD + Inhib group had an increase in carnitine and acetylcarnitine (Figure 4I and Supplemental Figure 8), while long-chain acylcarnitines, such as palmitoylcarnitine and stearoylcarnitine, were increased in the HFD + siRNA compared with the HFD + Inhib group (Figure 4J and Supplemental Figure 8). An increase in short-chain and a decrease in long-chain acylcarnitines is indicative of enhanced FAO in the HFD + Inhib group. In summary, the hepatic metabolome was profoundly different following KHK KD versus KHK inhibition. This difference is largely driven by the alterations in hepatic lipid handling.

### RNA-Seq analysis reveals profound and unique roles of KHK KD versus inhibition on the hepatic transcriptome.

**Figure 5:**
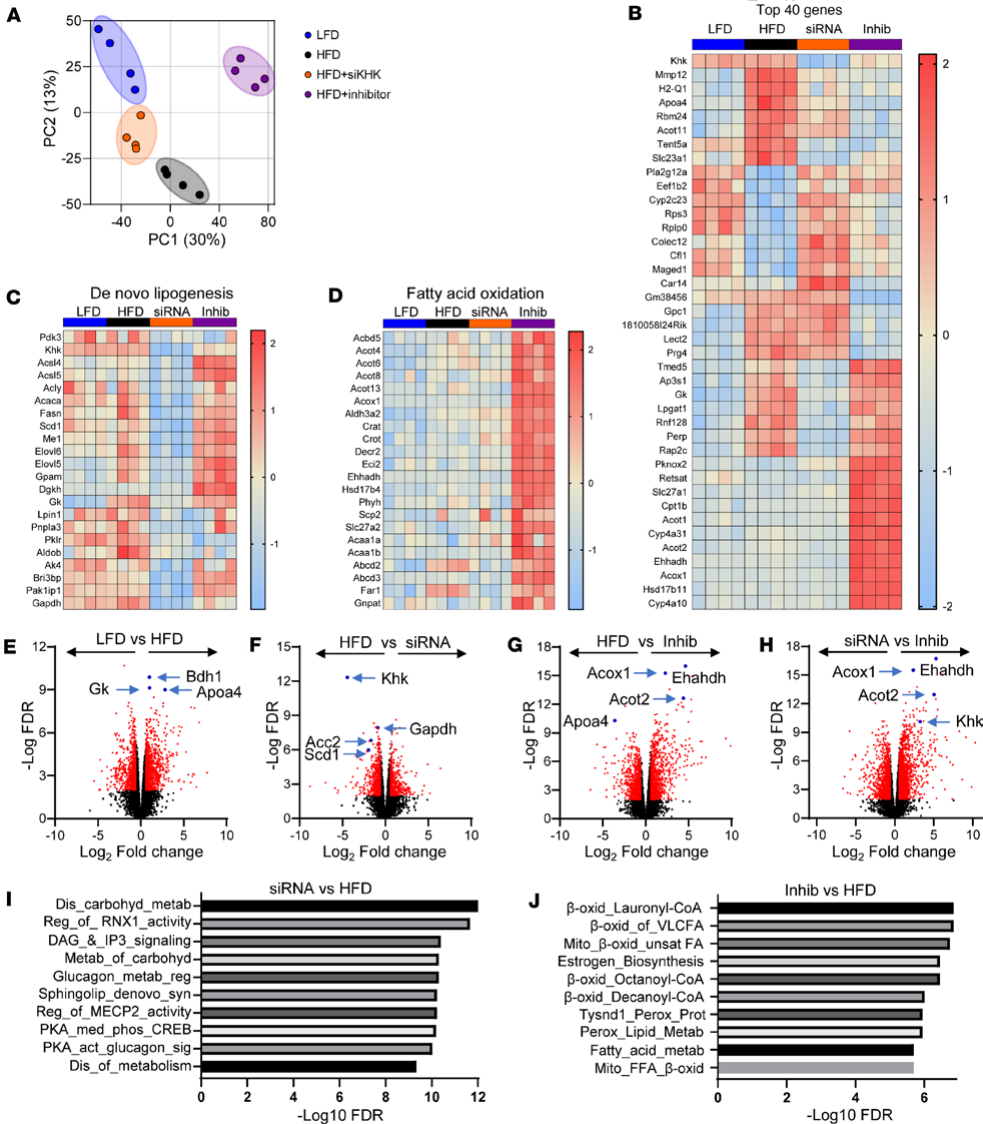
RNA-Seq analysis reveals profound and unique effects of KHK KD versus inhibition on hepatic transcriptome. (**A**) Principal component analysis of RNA-Seq data from the livers of our experimental mice. (**B**) A heatmap representation of the top 40 genes plus KHK. (**C**) Heatmap of the DNL pathway and (**D**) the FAO pathway. Volcano plot comparison of (**E**) HFD and LFD, (**F**) HFD and HFD + siRNA, (**G**) HFD and HFD + Inhibitor, and (**H**) HFD + siRNA and HFD + Inhibitor. Reactome pathway analysis showing the most significantly altered pathways between (**I**) HFD + siRNA versus HFD group and (**J**) HFD + Inhibitor versus HFD group.

We assessed global gene expression in these mice by RNA-Seq. The principal component analysis revealed that gene expression in the HFD group clustered differently than in the LFD group (Figure 5A). Following KHK KD, the gene expression clustered between the LFD and HFD groups, while KHK inhibition dramatically altered hepatic gene expression in a new direction. A heatmap analysis of the top 40 genes identified a unique pattern of gene expression among the groups (Figure 5B). Some of the genes that were upregulated in HFD and decreased by both KHK KD and inhibition are involved in the regulation of cholesterol, such as apolipoprotein A4 (Apoa4) and the formation of atherosclerotic plaques, such as matrix metalloproteinase 12. The genes that were decreased in HFD and restored by both KHK siRNA and inhibitor treatment are involved in ribosomal function, such as ribosomal protein S3 and ribosomal protein lateral stalk subunit P0. The genes that were lowered by KHK KD, but not by the inhibitor treatment, regulate lipid assembly, such as lysophosphatidylglycerol acyltransferase 1 and glycerol kinase (Gk). The genes that were normalized by the inhibitor, but not by KHK siRNA treatment, were members of proteoglycans, such as glypican 1 (Gpc1) and proteoglycan 4 (Prg4). Interestingly, a number of genes regulating FAO were increased uniquely in the inhibitor group, such as acyl-CoA thioesterase 2 (Acot2) and acyl-CoA oxidase 1 (Acox1), in agreement with our metabolomics analysis. Next, we honed in on the differentially regulated pathways. The expression of the DNL pathway, regulated by transcription factor carbohydrate-responsive element-binding protein (ChREBP), was profoundly downregulated by KHK siRNA treatment (Figure 5C). On the other hand, KHK inhibition led to upregulation of the FAO pathway, regulated by the PPARα transcription factor (Figure 5D).

Volcano plot comparison of the individual groups revealed that the upregulated genes in the HFD compared with the LFD group are involved in lipid homeostasis, including Gk, Apoa4, and 3-hydroxybutyrate dehydrogenase 1 (Figure 5E). The most downregulated genes in the KHK siRNA group, compared with the HFD group, were the genes involved in fructose metabolism, KHK and GAPDH, but also the genes involved in fatty acid synthesis, such as stearoyl-CoA desaturase 1 (Scd1) and acetyl-CoA carboxylase (Acc2) (Figure 5F). The genes increased with the inhibitor treatment, compared with the HFD group, are involved in lipid oxidation, including Acot2, Acox1, enoyl-CoA hydratase, and 3-hydroxyacyl CoA dehydrogenase (Figure 5G). These same genes were upregulated in the KHK + Inhib group compared with the HFD + siRNA group, as they were uniquely upregulated by the inhibitor treatment (Figure 5H). Reactome pathway analysis revealed that some of the most downregulated pathways in the HFD + siRNA group mediate carbohydrate metabolism and de novo synthesis of lipids, such as sphingolipids (Figure 5I). Conversely, some of the most upregulated pathways in the HFD + Inhib group were mediating mitochondrial and peroxisomal FAO (Figure 5J). These changes persisted when the HFD + Inhib was compared with the HFD + siRNA group (Supplemental Figure 9A).

An increase in FAO pathway and PPARα target genes with the inhibitor treatment was unexpected, so we assessed PPARα expression, which was elevated in the HFD and KHK + siRNA groups and was stepwise higher in the HFD + Inhib group (Supplemental Figure 9B). Next, we tested whether the inhibitor or F1P, produced by the inhibitor treatment, can bind to PPARα ligand binding domain to increase PPARα transcriptional activity in COS-7 cells transfected with PPARα luciferase reporter. As expected, WY14643, a PPARα agonist, increased luciferase activity in a dose-dependent fashion (Supplemental Figure 9C); however, neither the inhibitor (Supplemental Figure 9D) nor F1P (Supplemental Figure 9E) increased PPARα luciferase activity. Next, we performed kinase activity measures in real-time for serine/threonine kinases (STK) and phospho-tyrosine kinases (PTK) using PamGene PamStation microarray PamChip technology. Some of the most upregulated STKs in the HFD + siRNA, compared with the HFD group, were AKT1 and AKT2, in line with improved glucose tolerance in this group (Supplemental Figure 10A). Conversely, the most downregulated STK in the HFD + Inhib, compared with the HFD group, was glycogen synthase kinase 3β (GSK3β) (Supplemental Figure 10B). Lower GSK3β activity in the HFD + Inhib group may explain PPARα activation in this group, since GSK3β phosphorylates and deactivates PPARα. (35) Moreover, reduced GSK3β activity aligns with increased glycogen accumulation in this group, as GSK3β phosphorylates and deactivates GYS (36). There was no difference in PTK activity between KHK KD or inhibition (Supplemental Figure 10, C and D). In summary, KHK KD primarily lowered the DNL pathway, while the inhibitor treatment increased the FAO pathway.

### KHK KD decreased DNL, while the inhibitor increased the FAO pathway.

**Figure 6:**
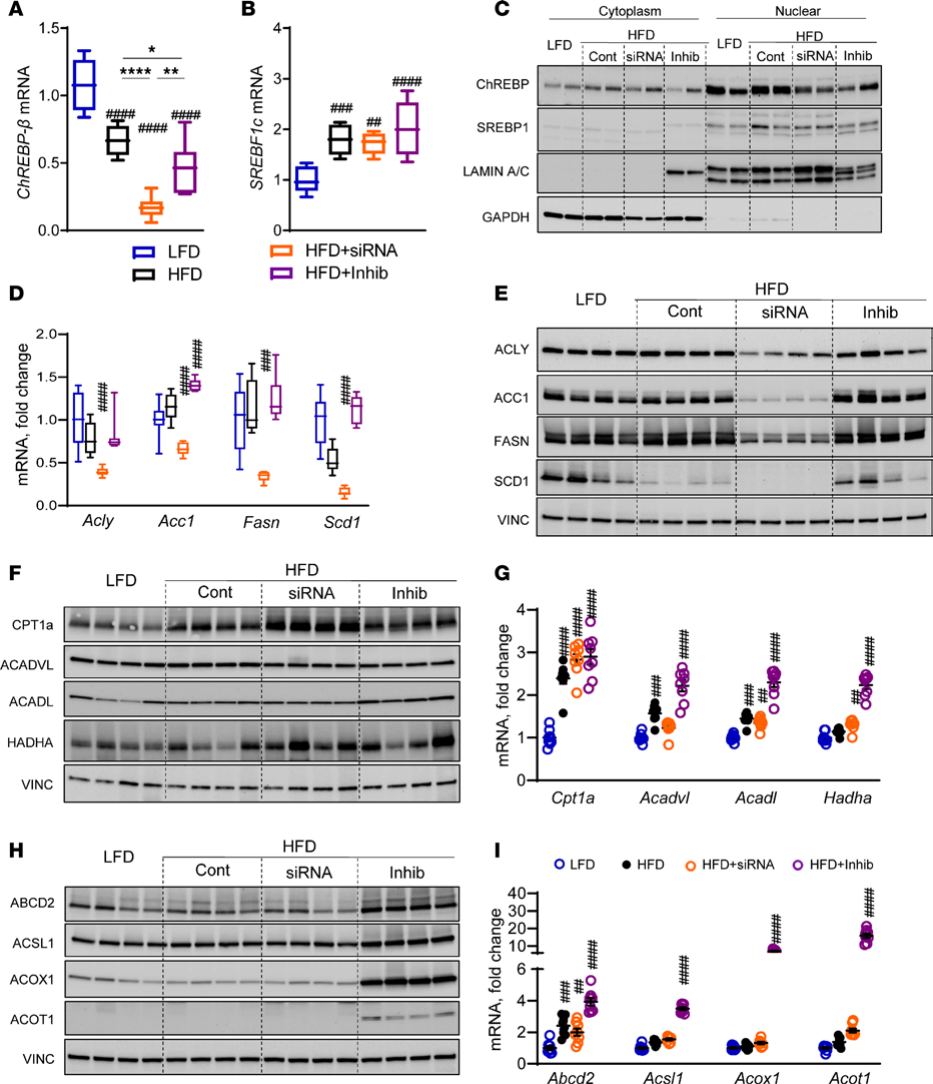
KHK KD lowers the DNL, while the inhibitor increases the FAO pathway. (**A**) ChREBP-β (MLXIPL-β) and (**B**) SREBF1c mRNA expression in the livers of our experimental mice. Box plots show the interquartile range, median (line), and minimum and maximum (whiskers). (**C**) Western blot showing nuclear translocation of ChREBP and SREBP1 proteins in the liver. (**D**) mRNA and (**E**) Western blot quantification of proteins involved in DNL. (**F**) Protein and (**G**) mRNA expression of genes regulating mitochondrial FAO. (**H**) Protein and (**I**) mRNA expression of genes regulating peroxisomal FAO. *n* = 7–8 mice per group for mRNA expression and *n* = 4 mice per group for protein quantification. Statistical analysis was performed using 1-way ANOVA compared with the LFD group (^##^*P* < 0.01; ^###^*P* < 0.001; ^####^*P* < 0.0001) with post hoc 2-tailed *t* tests between the individual groups (*P < 0.05; **P < 0.01; ****P < 0.0001).

We verified some of the most interesting changes in RNA-Seq data by qPCR and Western blot. ChREBP-β (MLXIPL-β), the active isoform of ChREBP transcription factor, was decreased in all mice on HFD, compared with the LFD group (Figure 6A), since the HFD contains fewer carbohydrates. MLXIPL-β was further reduced with the siRNA but not in the inhibitor group (Figure 6A). The expression of MLXIPL-α tended to be reduced in all mice on HFD (Supplemental Figure 11A). The expression of sterol regulatory element-binding transcription factor 1c (SREBF1c) was elevated 2-fold in all mice on HFD, compared with the LFD group, but there was no further effect with siRNA or the inhibitor treatment (Figure 6B). SREBF1a followed a similar expression pattern (Supplemental Figure 11B). Nuclear translocation of ChREBP was similar in mice on HFD and LFD (Figure 6C and Supplemental Figure 11C). KHK siRNA decreased ChREBP compared with the HFD group. The inhibitor treatment did not significantly lower ChREBP translocation when normalized to lamin. Nuclear translocation of SREBP1 was higher in mice on an HFD, compared with the LFD group (Figure 6C and Supplemental Figure 11D). SREBP1 was not markedly affected in the HFD + siRNA and HFD + Inhib groups. The expression of downstream genes mediating lipogenesis was not elevated in mice on an HFD but was profoundly reduced in the HFD + siRNA group, not in the HFD + Inhib group (Figure 6D), consistent with the changes in ChREBP. The protein levels of these lipogenic enzymes were likewise profoundly decreased only in the HFD + siRNA group (Figure 6E and Supplemental Figure 11E).

Next, we quantified the protein levels of the enzymes mediating mitochondrial β-oxidation. CPT1α, the rate-limiting enzyme of mitochondrial FAO, was increased in the HFD group compared with the LFD group (Figure 6F and Supplemental Figure 11F). CPT1α was further elevated in the HFD + siRNA, but not in the HFD + Inhib group. The protein levels of ACADVL, ACADL, and HADHA were not significantly affected. However, mRNA expression of these genes was significantly elevated in mice on an HFD, and they further increased in the HFD + Inhib but not in the HFD + siRNA group (Figure 6G). The protein levels of enzymes mediating peroxisomal FAO were markedly elevated 2- to 240-fold only in the HFD + Inhib group (Figure 6H and Supplemental Figure 11G) and their mRNA expression was 4- to 20-fold higher compared with the LFD group (Figure 6I). The expression of genes mediating fatty acid transport into the liver, CD36, Fatp2, and L-Fabp were also profoundly elevated in the HFD + Inhib group (Supplemental Figure 11H). In summary, KHK KD markedly decreased the DNL pathway, whereas the KHK inhibitor profoundly increased the FAO pathway, in agreement with RNA-Seq data.

### In vitro overexpression of WT, but not mutant kinase-dead KHK increases hepatocyte injury and glycogen accumulation.

**Figure 7:**
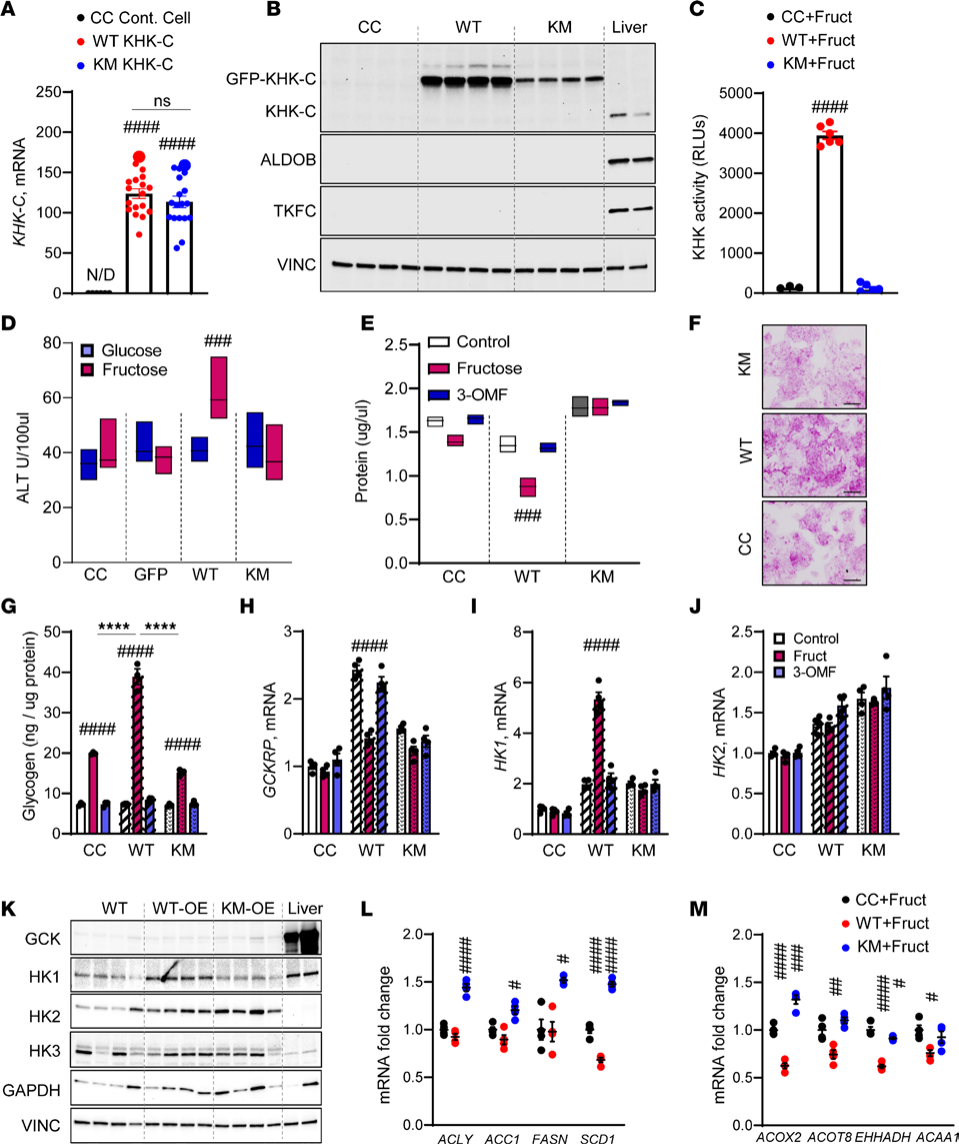
Overexpression of wild-type KHK supports glycogen accumulation, while overexpression of kinase-dead mutant KHK increases the expression of the DNL pathway. (**A**) KHK-C mRNA overexpression in control HepG2 cells, GFP-tagged wild-type mouse KHK-C–overexpressed cells (WT KHK-C), or mouse kinase-dead mutant–overexpressed (KM KHK-C) cells using lentivirus transfection. (**B**) Protein levels of fructose-metabolizing enzymes in control HepG2 cells, WT KHK-C cells, KM KHK-C cells, and mouse liver. (**C**) KHK activity in control HepG2 cells, WT KHK-C cells, and KM KHK-C cells treated with 5 mM fructose. (**D**) ALT level and (**E**) total protein after treatment with 5 mM fructose or 5 mM 3-O methylfructose (3-OMF) for 24 hours. (**F**) PAS staining of HepG2 cells treated with fructose for 24 hours. Scale bar, 10 µm. (**G**) Glycogen levels in control HepG2 cells, WT KHK-C cells, and KM KHK-C cells treated with fructose or 3-OMF. mRNA of Gckrp (**H**), Hk1 (**I**), and HK2 (**J**) expression in these cells treated with fructose or 3-OMF. (**K**) Western blot of enzymes involved in sugar metabolism in control HepG2 cells, WT KHK-C cells, KM KHK-C cells, and mouse liver. (**L**) mRNA expression of DNL genes and (**M**) FAO genes. *n* = 4 mice per group for gene expression and protein quantification. Statistical analysis was performed using 1-way ANOVA compared with the LFD group (^#^*P* < 0.05; ^##^*P* < 0.01; ^###^*P* < 0.001; ^####^*P* < 0.0001) with post hoc 2-tailed *t* tests between the individual groups (****P < 0.0001).

The differences between KHK KD and inhibition of its kinase activity may be secondary to the kinase-independent functions of KHK. We overexpressed GFP-tagged WT mouse KHK-C (WT) or kinase-dead mutant KHK-C (KM) in human HepG2 cells using lentivirus to test this hypothesis. KM KHK-C was generated by introducing a point mutation (G527R) in the ATP binding domain. For comparison, the KHK inhibitor similarly targets the ATP binding domain. Compared with the HepG2 control cells (CC), overexpression of both WT or KM robustly induced mouse KHK-C expression (Figure 7A). KHK-C protein was much higher in the cells overexpressing WT, compared with KM, suggesting lower protein stability (Figure 7B). Indeed, when treated with cycloheximide, a protein synthesis inhibitor, KM KHK-C protein degraded faster than WT KHK-C protein (Supplemental Figure 12A). KHK enzymatic activity was markedly elevated in WT KHK-C cells but not in KM KHK-C cells treated with fructose, verifying that the kinase-dead mutant is not able to metabolize fructose (Figure 7C). Increased KHK activity in cells that do not express downstream ALDOB or TKFC (Figure 7B and Supplemental Figure 12B) is expected to lead to the accumulation of F1P. Thus, WT KHK-C cells treated with 5 mM fructose, but not glucose, exhibited higher cell injury manifested by elevated ALT in cell supernatant (Figure 7D). Cell injury can lead to hepatocyte loss, and we saw lower total protein in WT KHK-C cells treated with fructose, but not 3-O methylfructose (3-OMF), a nonmetabolizable analog of fructose (Figure 7E). ALT and protein levels were not affected in KM cells.

Similar to in vivo findings, PAS glycogen staining was higher in fructose-treated WT but not KM cells or CC (Figure 7F). Indeed, glycogen levels were increased with fructose compared with glucose treatment in all cells but were the highest in the WT KHK-C cells (38.9 ± 2.0 ng/μg), compared with CC (19.9 ± 0.2 ng/μg) or KM (15.2 ± 0.5 ng/μg) cells (Figure 7G). HepG2 cells do not express Gck; however, Gckrp was increased over 2-fold in WT KHK-C cells, and it completely normalized following fructose, but not 3-OMF, treatment (Figure 7H). In the absence of Gck, Gckrp may regulate HK activity and expression. Indeed, Hk1 expression was 5-fold higher in WT KHK-C cells treated with fructose (Figure 7I). Hk2 expression was not affected by fructose or 3-OMF treatment (Figure 7J). In agreement with mRNA, GCK protein was not abundant in HepG2 cells compared with mouse liver (Figure 7K and Supplemental Figure 12C). HK1 protein was only elevated in WT KHK-C cells, whereas HK2 was increased 2- to 3-fold in both WT and KM cells (Figure 7K and Supplemental Figure 12D). HK3 was not altered, while GAPDH tended to be higher in both WT and KM cells.

Last, we interrogated DNL and FAO pathways. Fructose-treated WT KHK-C cells did not increase Acly, Acc1, Fasn, or Scd1 expression (Figure 7L). Interestingly, the cells overexpressing KM KHK-C had significantly higher expression of DNL enzymes. This suggests that the DNL pathway is, in part, regulated through the kinase-independent function of KHK-C. This correlated with increased expression of ChREBP and SREBP1c in KM but not WT cells (Supplemental Figure 12, E and F). Conversely, fructose-fed WT, but not KM, KHK-C cells had decreased expression of genes regulating FAO (Figure 7M). These data indicate that fructose metabolism and thus KHK activity are required to decrease FAO. In summary, KHK activity is required to generate F1P, induce glycogen accumulation, and decrease FAO, whereas DNL is independent of KHK-C kinase function.

## Discussion

In this work, we examined the impact of liver-specific KD of KHK versus systemic inhibition of KHK kinase activity. These 2 approaches targeting the same enzyme exert uniquely different metabolic outcomes. KHK siRNA completely and specifically deletes KHK protein in the liver. This leads to the upregulation of an alternative pathway of fructose metabolism via HK2 and improves glucose tolerance. Conversely, KHK inhibitor targets both KHK and TKFC proteins, resulting in F1P accumulation in the liver. Elevated F1P is associated with glycogen accumulation, hepatomegaly, and impaired glucose tolerance. Perhaps the most striking difference between KHK KD and inhibition is in the regulation of hepatic gene expression. KHK KD profoundly decreases the expression of genes in the DNL pathway, which is not observed following the inhibition of its kinase activity. Similarly, overexpression of kinase-dead mutant KHK-C increases the expression of DNL genes, suggesting a kinase-independent role. On the other hand, the KHK inhibitor uniquely upregulates the expression of PPARα target genes and alters hepatic metabolome in favor of elevated FAO. In conclusion, liver-specific KHK KD exerts beneficial effects on hepatic metabolism, while the inhibitor increases glycogen buildup via its off-target effect on TKFC. Therefore, siRNA-mediated KD of KHK may offer important advantages over the KHK inhibitor.

Liver-specific KHK KD induces a more complete abrogation of fructose metabolism, evident by minimal KHK activity, lower hepatic F1P/fructose ratio, and activation of an alternative pathway of fructolysis via HK2. Despite achieving adequate plasma IC_90_ concentration, fructose metabolism in the liver is only partially reduced by the inhibitor, as indicated by an incomplete reduction of KHK activity, normal F1P/fructose ratio, and no improvement in systemic glucose tolerance. The F1P/fructose ratio is higher in the inhibitor-treated mice than expected, given that partial inhibition of KHK activity should reduce fructolysis and, thus, F1P levels. Instead, F1P concentration was the highest in the HFD + Inhib group, which could be explained by lower TKFC activity. Consistent with this interpretation, Liu et al. reported increased F1P in primary hepatocytes treated with TKFC shRNA and fructose avoidance in TKFC-KO mice (37). A reduction in both KHK and TKFC activity may be due to the inhibitor targeting the ATP binding domain of these 2 enzymes. KHK and TKFC are the only kinases within the first 3 steps of fructose metabolism and thus possess ATP binding domain. Indeed, we show direct evidence that the KHK inhibitor is able to lower the enzymatic activity of recombinant mouse TKFC protein. We also show increased degradation of KHK-C protein harboring kinase inactivating mutation. A decrease in KHK and TKFC proteins could also be due to reduced ChREBP transcription of these enzymes. Contrary to this possibility, ChREBP is actually lower following KHK KD than with the inhibitor treatment. Taken together, these data implicate that the off-target effects of the KHK inhibitor on TKFC may, in part, explain F1P buildup and glycogen accumulation in the inhibitor-treated mice.

F1P is a toxic metabolite that leads to hepatic glycogen accumulation and hepatocellular injury (38). Cytotoxic properties of F1P have been documented from yeast (39) to human cells (40). We observe higher ALT and lower total protein in fructose-treated cells expressing WT KHK-C. Moreover, we also see increased glycogen accumulation in the livers of mice with elevated F1P and in fructose-treated cells overexpressing WT KHK-C. Glycogen accumulation in the HFD + Inhib mice can be accounted for by several mechanisms. Gck is bound by Gckrp that inhibits its activity (41). F1P promotes the dissociation of the complex and allows the production of substrates for glycogenesis (42). Additionally, F1P increases the activity of Gys2 (43). Another explanation for higher glycogen accumulation is a decrease in serum glucagon. These processes are interrelated as glucagon plays a role in regulating F1P levels (44).

The strong propensity of F1P to stimulate glycogen accumulation is observed in the cells that overexpress WT, but not kinase-dead KHK. These cells also lack ALDOB required for further metabolism of F1P. In vitro, fructose metabolism decreases Gckrp expression and leads to upregulation of HK1, which may provide a substrate for glycogen synthesis. Indeed, a dual role of F1P to support glycogenesis and induce hepatotoxicity is best observed in patients with hereditary fructose intolerance (HFI) due to ALDOB deficiency (38). The symptoms of HFI can be completely avoided in mice lacking KHK in order to prevent fructose metabolism to F1P (45). Interestingly, in our study, an increase in F1P following the inhibitor treatment induced glycogen accumulation but was not associated with increased liver injury or inflammation.

Both KD of KHK and inhibition of its kinase activity effectively resolve hepatic steatosis. However, these modalities reduce hepatic fat accumulation via substantially different mechanisms. RNA-Seq analysis reveals that a complete absence of KHK protein, but not inhibition of its kinase activity, decreases the expression of genes mediating DNL. This is verified by markedly lower mRNA and protein levels of DNL enzymes, Acly, Acc1, Fasn, and Scd1 in the HFD + siRNA but not the HFD + Inhib group. The effects on the DNL pathway are likely mediated by kinase-independent effects of KHK, as overexpression of kinase-dead mutant KHK was sufficient to increase the mRNA of DNL genes. We recently reported the first evidence pointing to kinase-independent effects of KHK in mediating protein acetylation (28). Acetylated proteins may not fold properly, triggering misfolded protein response and ER stress. Indeed, we reported that KHK drives ER stress, a function that is independent of fructose metabolism (29). Alternatively, the effects of KHK on the DNL pathway may be, in part, mediated by a greater reduction in ChREBP. Indeed, histone acetylation is a powerful mode of regulating gene expression, and acetylation of ChREBP increases its activity (46). Unlike our results, the KHK inhibitor has been reported to reduce DNL in rats (23). We may not have observed a decrease in the DNL pathway with the inhibitor treatment, as we did not use a diet high in fructose, we achieved only 38% inhibition of KHK in the liver, and mice are less sensitive to the effects of dietary fructose than rats. Most of our studies were preformed using an HFD, as this is the most common diet-induced obesity method, and a potential treatment for MASLD would ideally work for all patients, not only the ones consuming high amounts of fructose.

While KHK KD lowers the DNL pathway, the small molecule inhibitor uniquely and profoundly increases the expression of genes regulating FAO. Metabolomics analysis verified that the HFD + Inhib group had increased levels of short-chain and a decrease in long-chain acylcarnitines. Enhanced FAO pathway is not mediated by direct binding of the inhibitor to the PPARα ligand binding domain. However, lower GSK3β activity in the inhibitor group could activate the FAO pathway, as GSK3β has been reported to phosphorylate and inhibit PPARα (35). PPARα activation could also, in part, explain hepatomegaly in the HFD + Inhib group, as increased PPARα activity leads to hepatocyte proliferation (47). Moreover, PPARα activation explains an increase in cholesterol (48), which in mice mainly consists of HDL-cholesterol. Last, PPARα activation may account for lower serum glucagon since keto acids decrease glucagon secretion (49). In addition to PPARα activation, another mechanism for the enhanced FAO pathway is a decrease in TKFC. A KO of TKFC in the liver shifts the third step of fructose metabolism toward the ALDH pathway, resulting in increased FAO (37). In our study, ALDH was indeed more strongly upregulated in the HFD + Inhib than in the HFD + siRNA group. The inhibitor-induced upregulation of the FAO pathway may explain lower steatosis without a substantial decrease in the DNL pathway.

Fructose intake is a well-recognized risk factor for the development of obesity and metabolic dysfunction. We show that targeting KHK via liver-specific siRNA completely and specifically prevents fructose metabolism and results in the resolution of hepatic steatosis and improves glucose tolerance. On the contrary, a small molecule inhibitor of KHK only partially reduces KHK activity in the liver, but it also targets TKFC, resulting in elevated F1P, glycogen accumulation, hepatomegaly, and no improvement in glucose tolerance. Our study reaffirms that targeting KHK is still a viable option for the management of metabolic dysfunction in spite of the discontinuation of the KHK inhibitor from further clinical development. Based, in part, on the results of this study, our collaborators at Alnylam completed a phase I and are progressing with a phase II clinical trial (NCT05761301) testing KHK siRNA in patients with obesity and diabetes.

## Methods

### Sex as a biological variable

Our study exclusively examined male mice, as female mice do not develop profound obesity and fatty liver disease. It is unknown whether our findings are relevant to female mice.

### Animals and diets

Mice were housed at 20°C–22°C on a 12-hour light/12-hour dark cycle with ad libitum access to food and water. C57BL/6J male mice at 6 weeks of age were purchased from Jackson Laboratory and fed either an LFD (Research Diets D12450K) or a 60% HFD (Research Diets, D12492) for 6 weeks. Thereafter, the mice on an HFD were subdivided into 3 groups: the control group (HFD), the KHK siRNA group (HFD + siRNA), and the KHK inhibitor group (HFD + Inhib). To ensure equal stress/treatment, all mice were injected with either luciferase control siRNA (10 mg/kg) or siRNA targeting total KHK (20 mg/kg) every 2 weeks and were gavaged with either methylcellulose control or (15 mg/kg) KHK inhibitor (PF-06835919) in methylcellulose, twice daily for 4 weeks. The animals were weighed, and their food intake was recorded once per week. GTT and insulin tolerance test (ITT) were performed after 8 and 9 weeks of feeding, respectively. The mice were sacrificed after 10 weeks on the diet. The mice were injected with saline or 1 U of insulin (Humulin R-Lilly HI-213) via inferior vena cava 10 minutes before tissue collection in liquid nitrogen.

### Liver-specific KHK knockdown

Liver-specific knockdown was achieved by utilizing an siRNA conjugated to GalNAc. Alnylam Pharmaceuticals synthesized siRNA to specifically target mouse total KHK mRNA. siRNA has undergone chemical modifications to achieve long-lasting effect and specificity for hepatocytes. The guide strand is conjugated to a trivalent GalNAc specifically recognized by the asialoglycoprotein receptor, which is highly expressed on the surface of hepatocytes, achieving hepatocyte-specific delivery and uptake.

### KHK inhibitor dose calculations and pharmacokinetic studies

KHK inhibitor PF-06835919 was synthesized at Pharmaron based on the structure described (50). The in vitro potency, 5 nM, of the compound was confirmed using the method described (32). The compound was formulated in 0.5% HPMC 10,000 cP, 0.1% Tween 80, pH 9.

Male C57BL/6J mice on 60% HFD (Research Diets D12492) starting at 6 weeks of age were purchased at 16 weeks of age from Taconic Denmark and kept in an Association for Assessment and Accreditation of Laboratory Animal Care International–accredited facility. Prior to the fructose tolerance test, the mice were gavaged with 10 mg/kg, 30 mg/kg, or 60 mg/kg of the KHK inhibitor or methylcellulose vehicle. One hour later, 6 mg/kg of fructose or water was gavaged, and repeated blood samples were collected over 1 hour. Fructose was analyzed using BioAssay Systems EnzyChrom Fructose assay kit (EFRU-100), glucose was measured using ACCU-chek device, and insulin was measured by ELISA (Crystal Chem Ultra Sensitive Mouse Insulin ELISA Kit (catalog 90080).

#### In vivo inhibitor concentration.

Plasma was collected for exposure analysis at the termination of the main experiment. The plasma samples were precipitated with 100% acetonitrile. After vortexing and centrifugation, the supernatant was transferred to a new plate and diluted 1:2 with H_2_O, 0.2% formic acid. Matrix-matched calibration samples, quality controls for PF-06835919, and blanks were prepared in the same way as the study samples. The samples were injected on an Acquity Ultra Performance liquid chromatographer coupled to a Sciex API 4500 mass spectrometer. The MS method was operated in positive-electrospray mode using multiple reaction monitoring of transitions 357.2 > 315.3 for PF-068359198. Plasma concentration of PF-06835919 was measured from 2 to 5 hours after the last dose. Therefore, we fitted these data with 1 compartmental pharmacokinetic model to assess if the average plasma concentration was in line with the predicted target exposure.

### Glucose and insulin tolerance test

For GTT, mice were fasted overnight and injected i.p. with 2 g glucose/kg body mass. Blood glucose levels were measured at 0, 15, 30, 60, and 120 minutes using a glucose meter (Infinity, US Diagnostics). ITTs were performed in nonfasted mice by i.p. injection of 1 mU insulin/kg body mass. Blood glucose levels were measured at indicated times.

### Liver histology and serum assays

The slides were prepared from formalin-fixed, paraffin-embedded liver sections. H&E staining and PAS staining were performed by the University of Kentucky Pathology Research Core. Liver homogenates were used for the quantification of triglycerides (Pointer Scientific, T7532-1L) following the manufacturer’s guidelines, as previously published (51). Plasma insulin was quantified using an ELISA kit (Crystal Chem, 90080). ALT levels were measured using commercial ALT kit (Catachem, C164-0A). Urinary fructose was quantified using a fructose assay kit (BioAssay Systems, EFRU-100).

### qPCR and mRNA quantification

Gene expression was quantified as previously described (52). Briefly, mRNA was extracted by homogenizing liver tissue in TRIzol (Invitrogen), treating with chloroform, and precipitating in 70% ethanol. mRNA was purified using RNeasy Mini Kit columns (QIAGEN, 74106). cDNA was made using High-Capacity cDNA Reverse Transcription Kit (Applied Biosystems, 4368813). qPCR was performed utilizing C1000 Thermal Cycler (Bio-Rad, CFX384) and QuantStudio 7 Flex Real-Time PCR System (Thermo Fisher Scientific, 4485701). Mouse and human primer sequences are listed in Supplemental Tables 1 and 2, respectively. An average of 18S and TBP was used to normalize the mRNA data.

### Protein extraction and immunoblot

Tissues were homogenized in RIPA buffer (MilliporeSigma) with protease and phosphatase inhibitor cocktail (Bimake.com, B14002, B15002). A total of 10–20 μg of protein was loaded on the gel. Proteins were separated using SDS-PAGE gel and transferred to the PVDF membrane (MilliporeSigma). Immunoblotting was achieved using the indicated antibodies listed in the Supplemental Table 3. Images were captured by ChemiDoc MP imaging system (Bio-Rad, 12003154) and iBright imaging system (Thermo Fisher Scientific, CL1000). Quantification of immunoblots was performed using ImageJ (NIH).

### KHK and TKFC activity assays

KHK activity was measured with luminescence-based method as previously published (32). TKFC activity was measured using mouse recombinant TKFC protein (Origene, TP509059). The assay measures ADP production in a reaction where GA and ATP are converted by d-glyceraldehyde-3-phosphate and ADP. Recombinant TKFC protein was preincubated with KHK inhibitor or vehicle control for 15 minutes at 37°C. Thereafter, the mixture was added to the reaction containing 100 mM Tris-HCl, 5 mM MgCl_2_, 200 μM ATP, and 0.1 mg/mL bovine serum albumin at pH 7.5 and incubated for 5 minutes at room temperature. Then, 0.5 mM GA was added and incubated for an additional 60 minutes at 37°C. ATP consumption was measured using an ADP-Glo kit (Promega, PAV6930).

### Generation of stable KHK-C–overexpressing HepG2 cells

HepG2 cells were purchased from American Type Culture Collection and cultured under standard conditions in 1:1 DMEM and Ham’s F12 media (Corning) supplemented with 10% fetal bovine serum and 1% penicillin-streptomycin. Lentiviral particles were purchased from Origene containing the mouse KHK-C sequence (MR204149L2V) or kinase-dead mutated KHK-C fused to GFP (custom ordered, Origene). HepG2 cells were transduced with lentivirus at an MOI of 5, and media were changed every 2 days. After 5 days, GFP^+^ cells were sorted and propagated for an additional 2–3 weeks. KHK-C was quantified by both qPCR and Western blot.

### Matrix-assisted laser desorption/ionization mass spectrometry imaging of glycogen in situ

Matrix-assisted laser desorption (MALDI) imaging of in situ glycogen was performed based on the previously described technique (53). Formalin-fixed, paraffin-embedded tissues or purified glycogen were sectioned at 5 μm or spotted directly onto glass slides. The slide went through dewaxing and rehydration washes, followed by antigen retrieval in a citraconic anhydride buffer solution. An HTX M5 robotic sprayer was used to coat tissues in a solution of isoamylase. Following the application of enzyme, the slides were incubated for 2 hours at 37°C in a humidity chamber, then dried overnight in a vacuum desiccator. The following day, the robotic sprayer coated the slides with a 7 mg/mL CHCA matrix, and the slides were stored in a desiccator until MALDI–ionization mass spectrometry analysis. The slides were loaded into a Waters Synapt G2 SX mass spectrometer that used a UV laser of 100 μm in size to detect N-glycans and glycogen. Data analysis was then performed using Waters High Definition Imaging software.

### RNA sequence analysis and bioinformatics methods

HTG EdgeSeq mRNA sequence analysis was performed by the BioPolymers Facility at Harvard Medical School. Bioinformatics analysis was performed as we previously published (54).

### PPARα transcriptional activity assay

See Supplemental Methods.

### Metabolomics and kinome analysis

See Supplemental Methods.

### Statistics

All data are presented as the mean ± SEM. The data analysis, comparing the effects of control with experimental conditions, was first performed using 1-way ANOVA with Dunnett’s multiple comparisons test for comparison of the individual groups. Significant differences among control and experimental groups are noted with a number sign (#), and significant differences between the individual groups under a black line are indicated by an asterisk (*). For both signs, a single symbol represents a *P* < 0.05; 2 symbols represent a *P* < 0.01; 3 symbols denote a *P* < 0.001, and 4 symbols represent a *P* < 0.0001 throughout the study. *P* < 0.05 was considered statistically significant.

### Study approval

All animal protocols were in accordance with the *Guide for the Care and Use of Laboratory Animals* (National Academies Press, 2011) and were approved by the IACUC of the University of Kentucky. For the pilot fructose tolerance test, the experimental procedures were approved by the Gothenburg, Sweden, ethics review committee on animal experiments (2002–2019).

### Data availability

The RNA-Seq data sets can be accessed from NCBI Gene Expression Omnibus with accession ID GSE278322. Metabolomics analysis is included in Supplemental Table 4. Values for all data points shown in graphs can be found in the Supporting Data Values file.

## Author contributions

SHP and SS conceived the study; LRC, HAC, RCS, KW, GO, JB, and TDH developed methodology; LRC, RCS, ZAK, EAB, SJ, and CJ performed formal analysis; SHP, TF, LRC, HAC, RCS, KW, JB, GO, AB, MP, SJ, ASL, CJ, ZAK, EAB, TDH, and GJM investigated; KW, GO, JB, SD, TDH, and CJ provided resources; LRC, RCS, ZAK, EAB, and SJ curated data; SHP and SS wrote the original draft; SS supervised; SHP, TF, and SS performed project administration; and SS acquired funding.

## Supplementary Material

Supplemental data

Unedited blot and gel images

Supplemental table 1

Supplemental table 2

Supplemental table 3

Supplemental table 4

Supporting data values

## Version 1 10/17/2024

In-Press Preview

## Version 2 12/06/2024

Electronic publication
