# Supplementary material for: Knockdown of ketohexokinase versus inhibition of its kinase activity exert divergent effects on fructose metabolism
Source: JCI Insight. 2024 Dec 6;9(23):e184396. doi: 10.1172/jci.insight.184396 (PMC11623947; doi:10.1172/jci.insight.184396)
Supplement: Supplemental data [file jciinsight-9-184396-s170.pdf]

PPARα Transcriptional Activity Assay

We measured PPARα transcriptional activity as previously described in (1). In brief, we transiently transfected Cos7 cells using the Neon™ Transfection System per the manufacturer's settings, with a plasmid containing the 9x upstream activation sequence (9xUAS) GAL4-driven promoter luciferase vector construct (9x UAS-Luc, 16μg) along with a plasmid containing the GAL4 DNA-binding domain fused with the LBD of PPARα (GAL4-PPARα-LBD, 6μg), and a pRL-CMV plasmid for Renilla luciferase expression (0.25μg) as a transfection normalizing control. 24 hours after electroporation, warm DMEM + 10% dialyzed FBS + 1% AA was added to the cells. After an additional 24 hours, the cells were treated with increasing doses of WY14643 (0.125 - 50 μM), KHKi (0.125 - 10 μM), Fructos-1-Phosphate (100 nM - 1mM), and Fructos-1-Phosphate (100 nM - 1mM) + 25μM WY14643 for 24 h. The activity was then measured by dividing Firefly luciferase with the Renilla luciferase using the Dual-Luciferase® reporter assay system (Promega) with the Promega GloMax® 96 microplate luminometer.

Metabolomics and kinome analysis

Mass spectrometry analysis quantified metabolome from the livers of mice in Dr. Jang laboratory as previously published (2). Metabolites in the liver were extracted by ice-cold acetonitrile:methanol:water (40:40:20) solution containing 0.5% formic acid. 15% NH4HCO3 (w/v) in acetonitrile:methanol:water solution was added to neutralize pH. Metabolites were analyzed by quadrupole-orbitrap mass spectrometer (Q-Exactive Plus Hybrid Quadrupole-Orbitrap, Thermo Fisher) mass spectrometers coupled to hydrophilic interaction chromatography (HILIC) via electrospray ionization. LC separation was on an Xbridge BEH amide column at 25°C using a gradient of solvent A (5% acetonitrile in water with 20 mM ammonium acetate and 20 mM ammonium hydroxide) and solvent B (100% acetonitrile). Flow rate was 350 μL/min. The LC gradient was: 0 min, 75% B; 3 min, 75% B; 4 min, 50% B; 5 min,

10% B; 7 min, 10% B; 7.5 min, 75% B; 11 min, 75% B. MS analysis were acquired in negative ion mode with MS Full-scan mode from m/z 70 to 830 and 140,000 resolution. Data were analyzed using the Compound Discoverer (Thermo Fisher Scientific, San Jose, CA) software for automated peak picking.

Serine/threonine kinase (STK) and phospho-tyrosine kinase (PTK) kinome analysis was measured using PamGen PamStation microarray chip technology as previously described (3). Upstream kinase analysis (UKA) was performed using BioNavigator (PamGene) as previously described in (4-6).

References:

1. Gordon DM, Neifer KL, Hamoud AA, Hawk CF, Nestor-Kalinoski AL, Miruzzi SA, et al. Bilirubin remodels murine white adipose tissue by reshaping mitochondrial activity and the coregulator profile of peroxisome proliferator-activated receptor alpha. *J Biol Chem.* 2020;295(29):9804-22.
2. Jang C, Wada S, Yang S, Gosis B, Zeng X, Zhang Z, et al. The small intestine shields the liver from fructose-induced steatosis. *Nat Metab.* 2020;2(7):586-93.
3. Creeden JF, Kipp ZA, Xu M, Flight RM, Moseley HNB, Martinez GJ, et al. Hepatic kinome atlas: An in-depth identification of kinase pathways in liver fibrosis of humans and rodents. *Hepatology.* 2022.
4. Bates EA, Kipp ZA, Martinez GJ, Badmus OO, Soundarapandian MM, Foster D, et al. Suppressing Hepatic UGT1A1 Increases Plasma Bilirubin, Lowers Plasma Urobilin, Reorganizes Kinase Signaling Pathways and Lipid Species and Improves Fatty Liver Disease. *Biomolecules.* 2023;13(2).
5. Badmus OO, Kipp ZA, Bates EA, da Silva AA, Taylor LC, Martinez GJ, et al. Loss of hepatic PPARalpha in mice causes hypertension and cardiovascular disease. *Am J Physiol Regul Integr Comp Physiol.* 2023;325(1):R81-R95.
6. Zelows MM, Cady C, Dharanipragada N, Mead AE, Kipp ZA, Bates EA, et al. Loss of carnitine palmitoyltransferase 1a reduces docosahexaenoic acid-containing phospholipids and drives sexually dimorphic liver disease in mice. *Mol Metab.* 2023;78:101815.

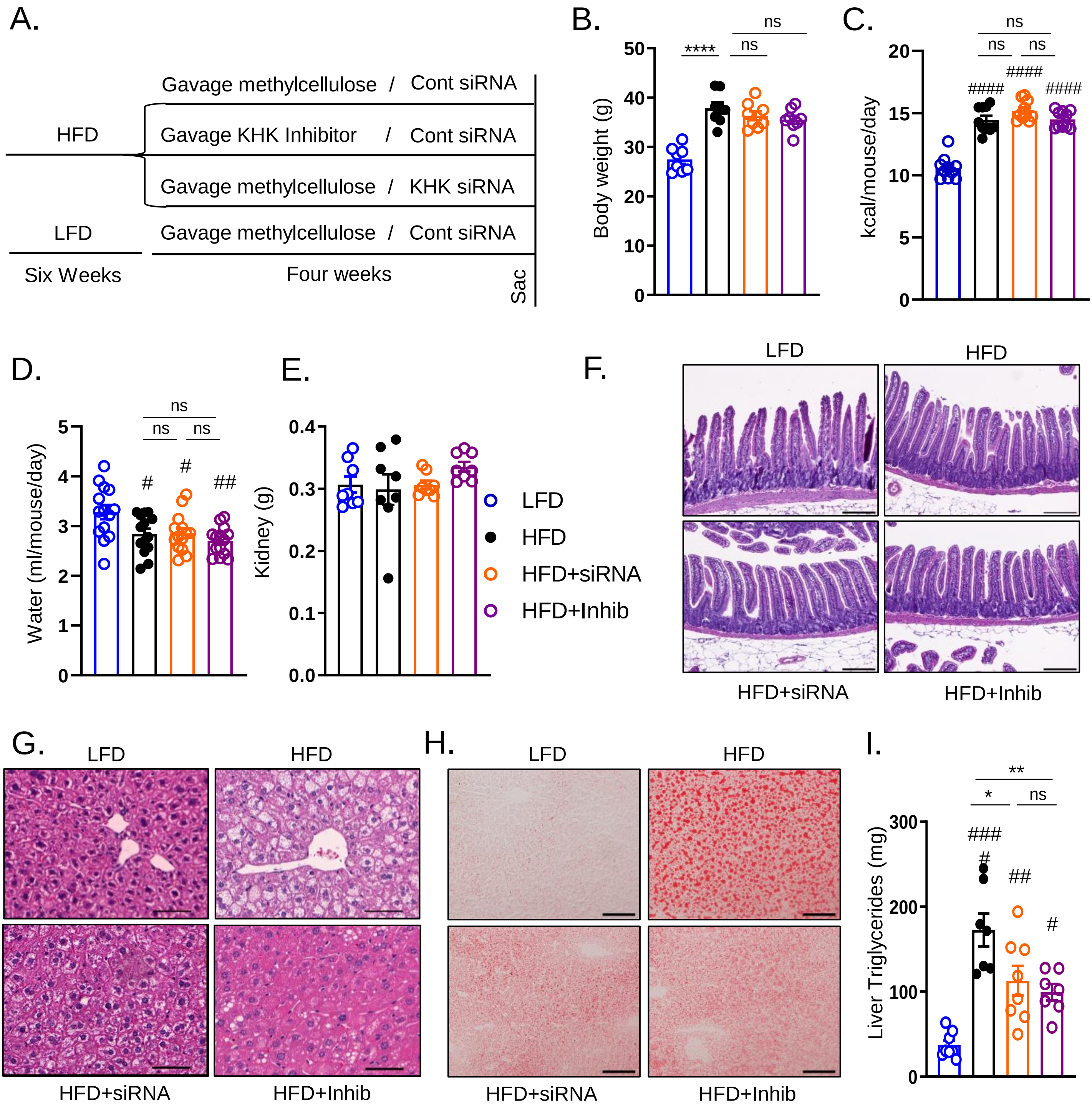

Supplemental Figure 1: The effects of KHK siRNA and inhibitor on anthropometric measures and liver physiology

A) Graphical representation of the study design. B) Body weight in these mice at the end of the study. C) Caloric intake and D) water consumption measured per cage once weekly for the duration of the experiment and averaged per mouse per day. E) Kidney weight at the time of the sacrifice. F) Representative H&E images of intestinal histology. Scale bar=200µm, G) Representative H&E images of liver histology. Scale bar=50µm. H) Representative Oil Red-O stain of liver sections. Scale bar=50µm. I) Triglyceride accumulation in the livers of these mice. n = 7-8 mice per group. The bar graphs show mean ±SEM. # represents statistically significant results by one way ANOVA compared to the LFD group. * represents post-hock t-test between the groups.

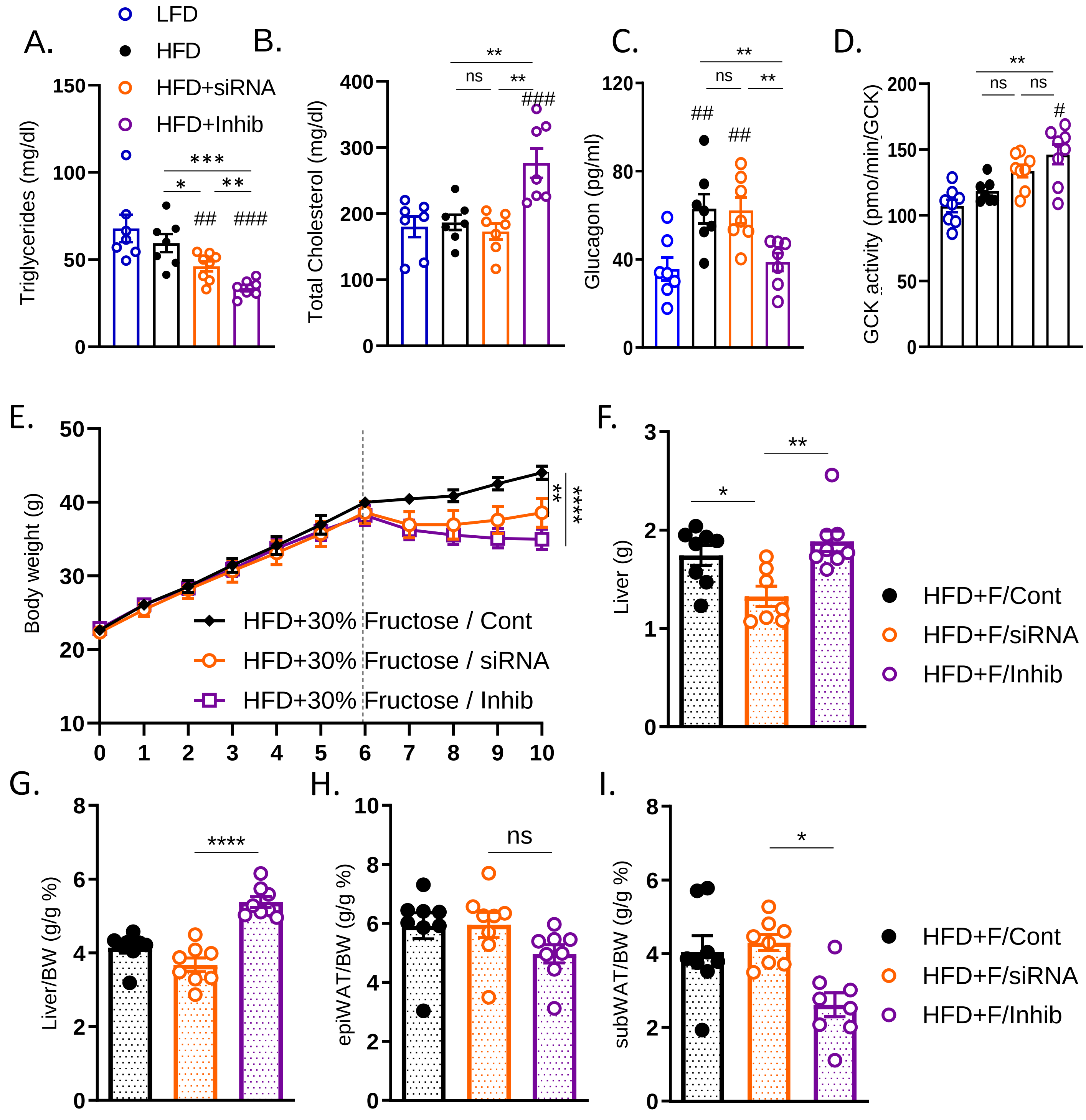

Supplemental Figure 2: The effects of KHK siRNA and inhibitor in mice on high-fat, high-sugar diet

A) Serum triglycerides and B) total cholesterol at sacrifice in mice on LFD or HFD treated with KHK siRNA or the inhibitor. C) Serum glucagon level measured between 9-11am. The mice were fasted for 2h. D) GCK activity in liver lysates from these mice. E) Body weight in mice on 60% HFD supplemented with 30% fructose in drinking water and treated with KHK siRNA or the inhibitor. F) Liver weight of these mice at sacrifice. G) Liver mass normalized by body weight. H) Epididymal white adipose tissue (epiWAT) mass normalized by body weight, and I) Subcutaneous white adipose tissue (subWAT) mass normalized by body weight. n = 8 mice per group. The bar graphs show mean ±SEM. * represents statistically significant results by ordinary one-way ANOVA with Bonferroni multiple comparisons post-test compared to the HFD+F/siRNA group.

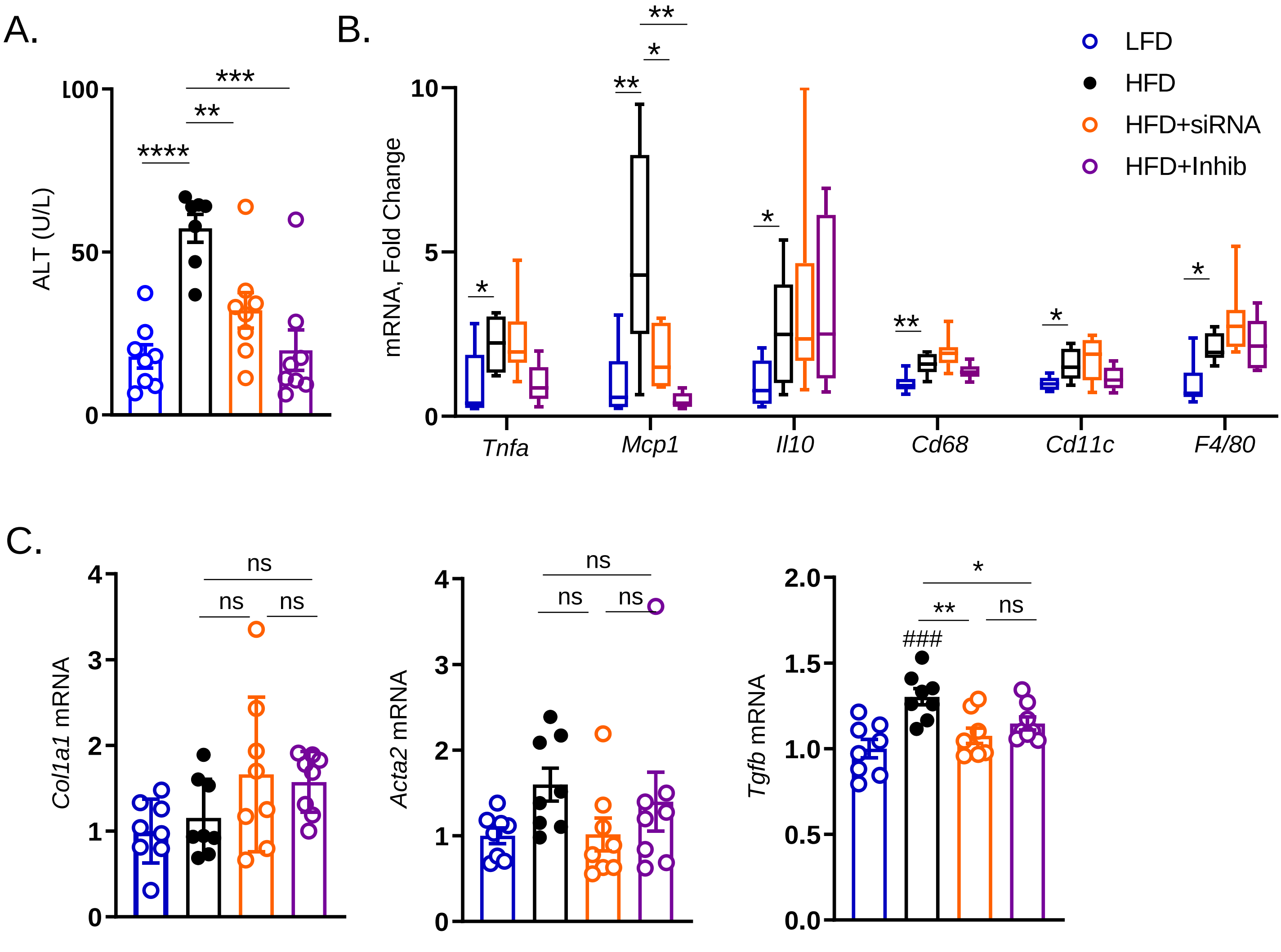

Supplemental Figure 3: The effects of KHK siRNA v inhibitor on the markers of liver injury

A) Serum ALT at the time of the sacrifice. B) Hepatic mRNA expression of inflammatory genes in these mice. n = 6 mice per group. C) mRNA expression of genes regulating liver fibrosis. n= 8 mice per group. The bar graphs show mean ±SEM. # represents statistically significant results by one-way ANOVA compared to the LFD group. * represents post-hock t-test between the groups.

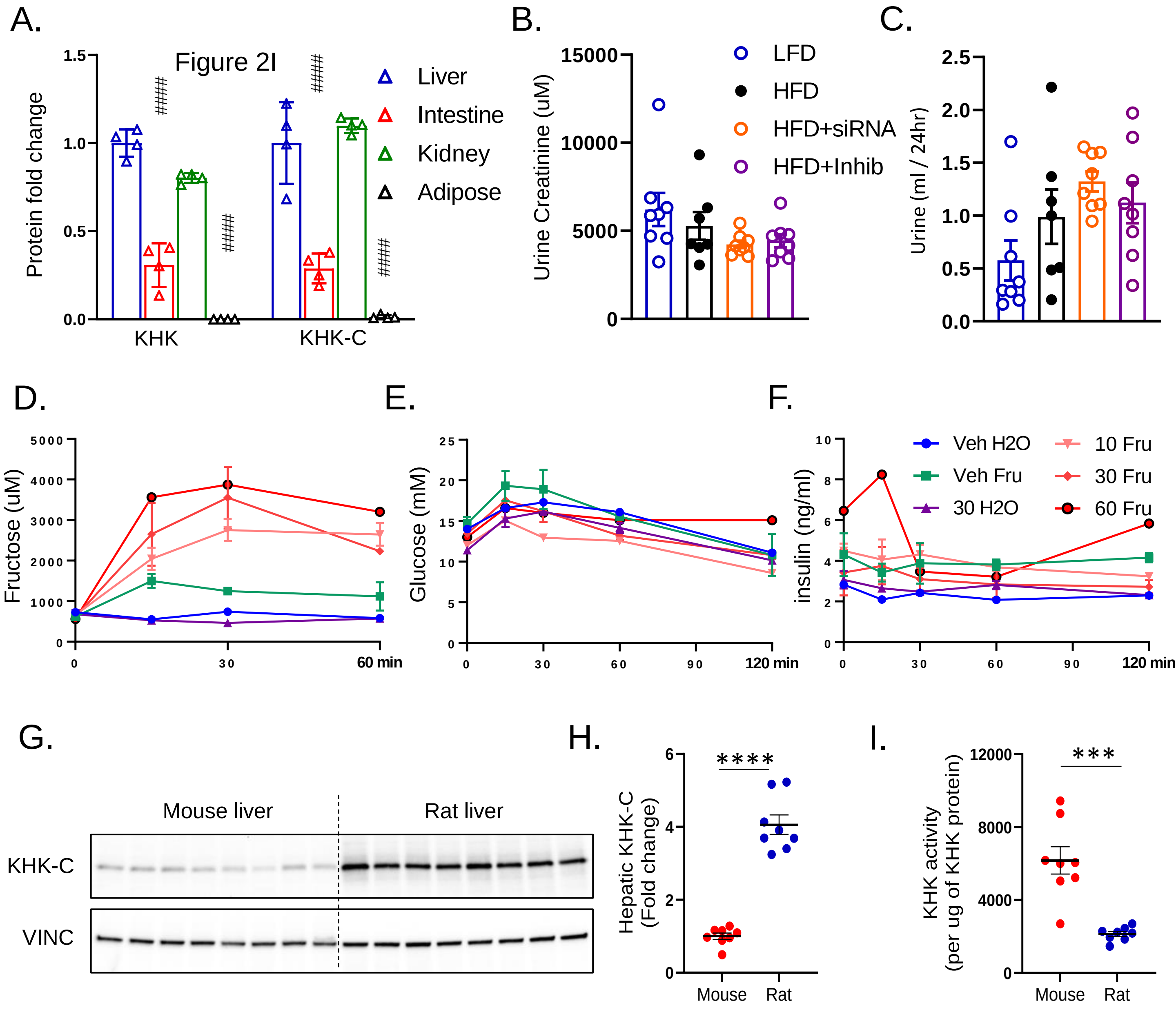

Supplemental Figure 4: Protein quantification, urine collection, acute effects of the inhibitor and KHK-C protein in mice and rats.

A) Densitometry quantification of western blot data in figure 2I using Image J. n = four mice per group. B) Urine creatinine concentration and C) Urine volume collected over 24h between 9-10 weeks on the diet. n= 7-8 mice per group. The bar graphs show mean ±SEM. # represents statistically significant results by one-way ANOVA compared to the LFD group. * represents post-hock t-test between the groups. Serum D) fructose, E) glucose and F) insulin levels in chow-fed C57Bl/6J male mice gavaged with 10 mg/kg, 30 mg/kg or 60 mg/kg of the ketohexokinase inhibitor or methylcellulose vehicle one hour before 6 mg/kg fructose or water gavage. n = 1-2 mice per group. The data points represent mean ±SEM. G) Western blot quantification of KHK-C from the livers of male C57Bl6/J mice and Sprague Dawley rats fed a chow diet. H) Image J quantification of KHK-C western blot data I) KHK enzymatic activity determined from the livers of these mice and rats. n = 8 mice or rats per group. * represents t-test between the groups.

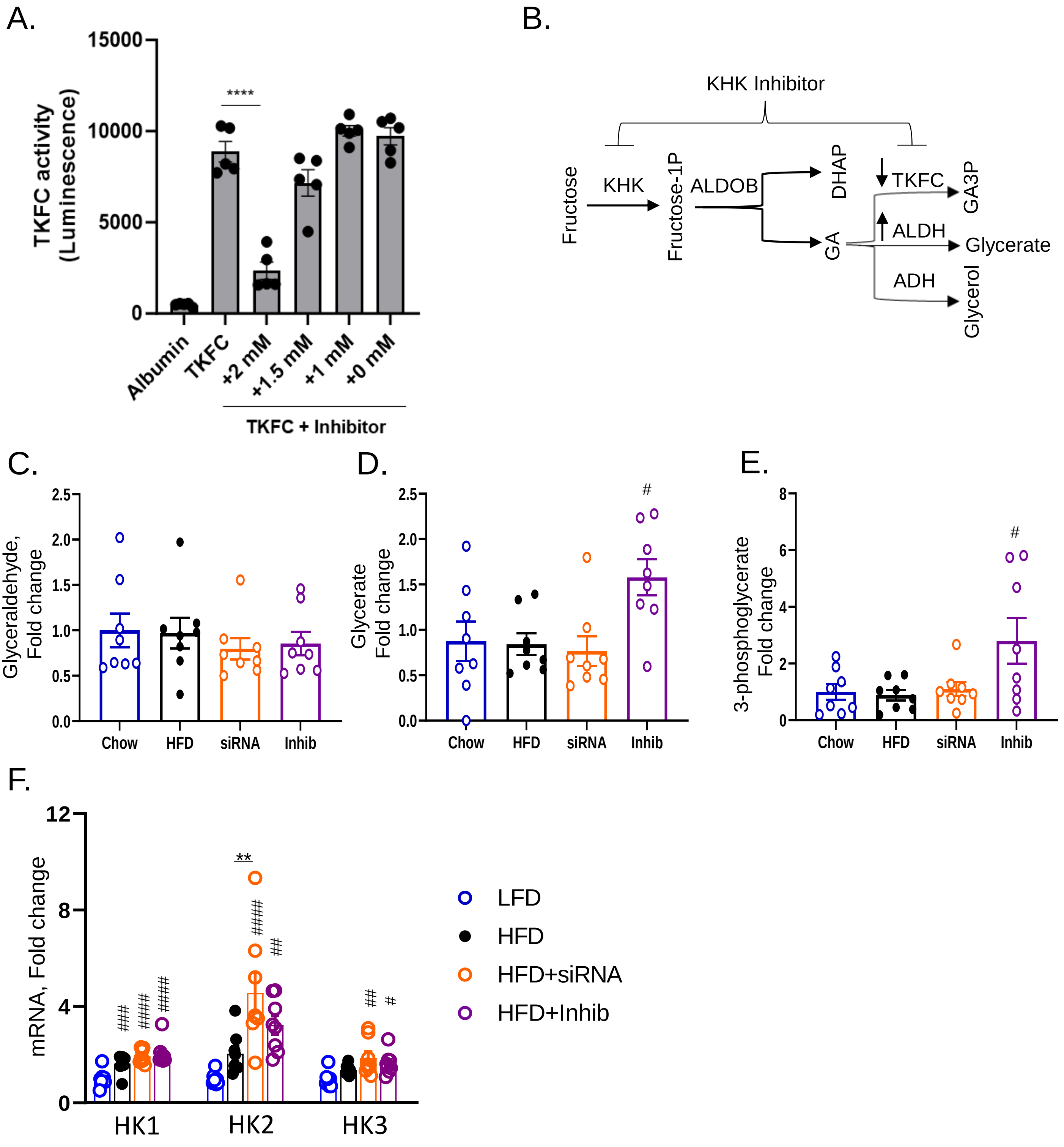

Supplemental Figure 5: TKFC activity and expression of hexokinases.

A) TKFC enzymatic activity determined using 50 ng of recombinant mouse TKFC protein treated with different concentrations of KHK inhibitor. n = 5 replicate reactions per group. B) The fructolysis pathway. Fructose-1P = fructose 1 phosphate; GA = Glyceraldehyde; GA3P = Glyceraldehyde-3 phosphate. The levels of C) glyceraldehyde, D) glycerate and E) 3-phosphoglycerate in the liver at the end of the ten-week experiment quantified by MS. F) Hepatic mRNA expression of hexokinase isoforms from the livers obtained at sacrifice. n = 6 mice per group. The bar graphs show mean ±SEM. # represents statistically significant results by one-way ANOVA compared to the LFD group. * represents the post-hock t-test between the groups.

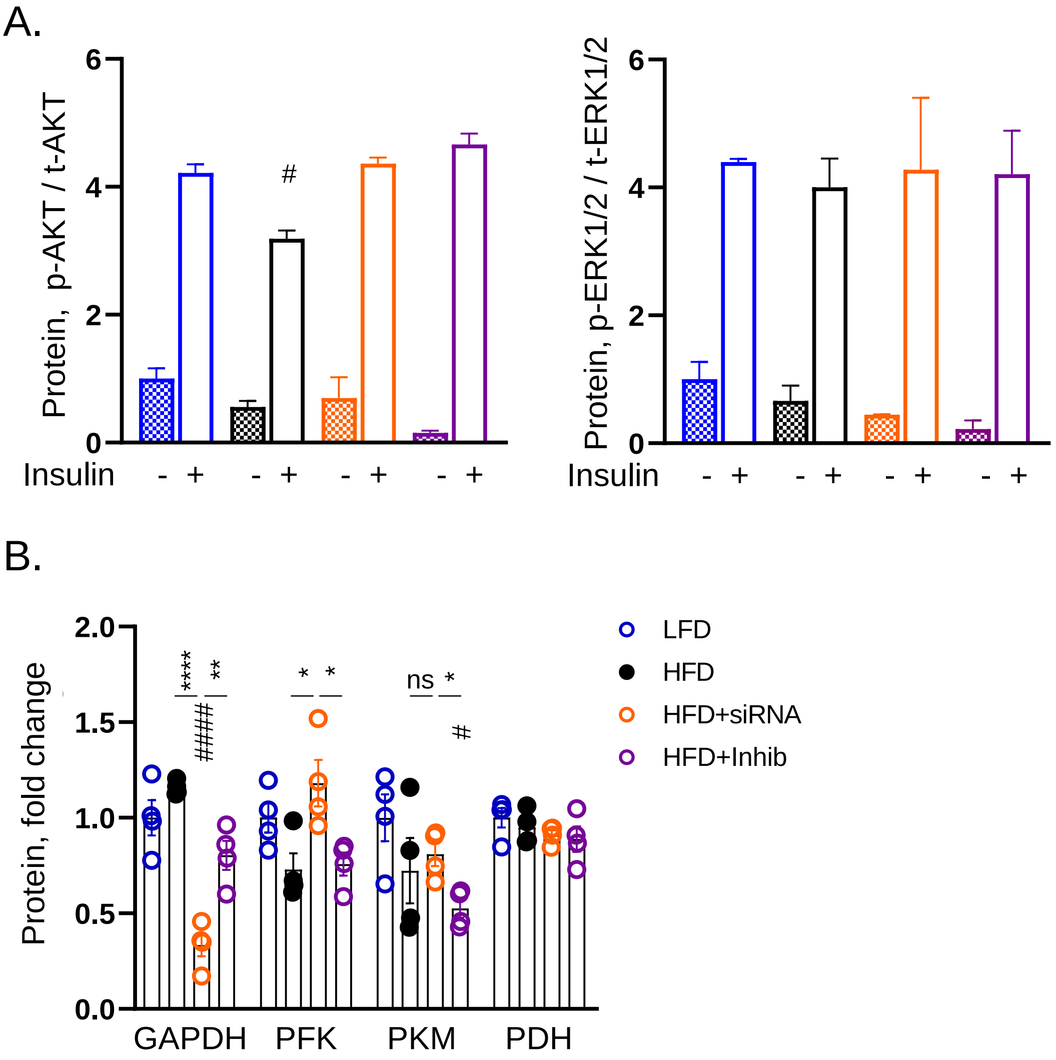

Supplemental Figure 6: Western blot protein quantification

A) Densitometry quantification of western blot pAkt and pERK data in figure 4F using Image J. n = four mice per group. The bar graphs show mean ±SEM. # represents statistically significant results by one-way ANOVA comparing insulin treated groups to the LFD group. B) Western blot quantification of figure 4G using image J. n = 4 mice per group. The bar graphs show mean ±SEM. # represents statistically significant results by one-way ANOVA compared to the LFD group. * represents post-hock t-test between the groups.

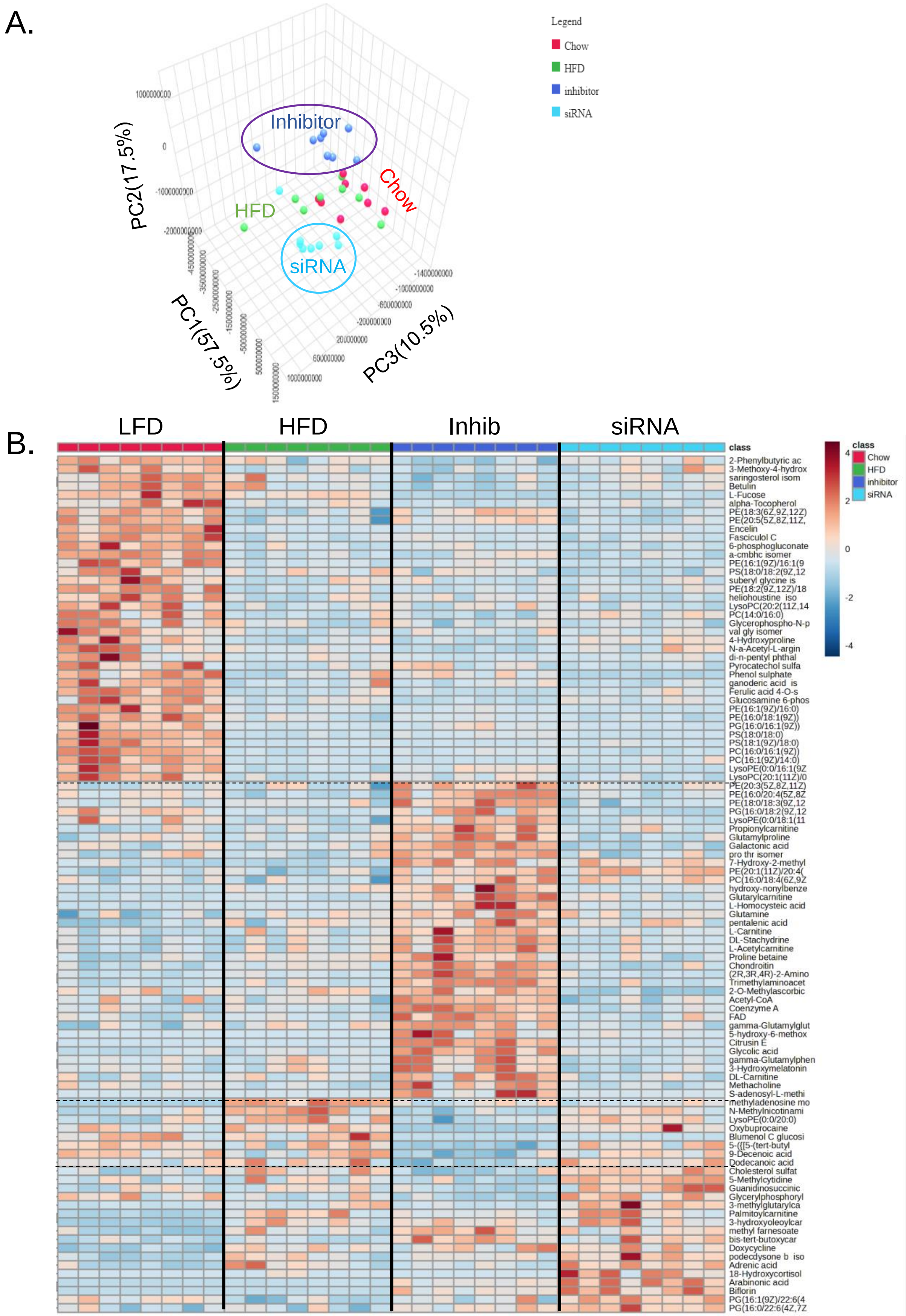

Supplemental Figure 7: Mass spec analysis of liver metabolites.

A) Principal component analysis of 880 hepatic metabolites quantified by mass spectrometry and B) Heatmap representation of 110 most significantly altered metabolites. n = 8 mice per group.

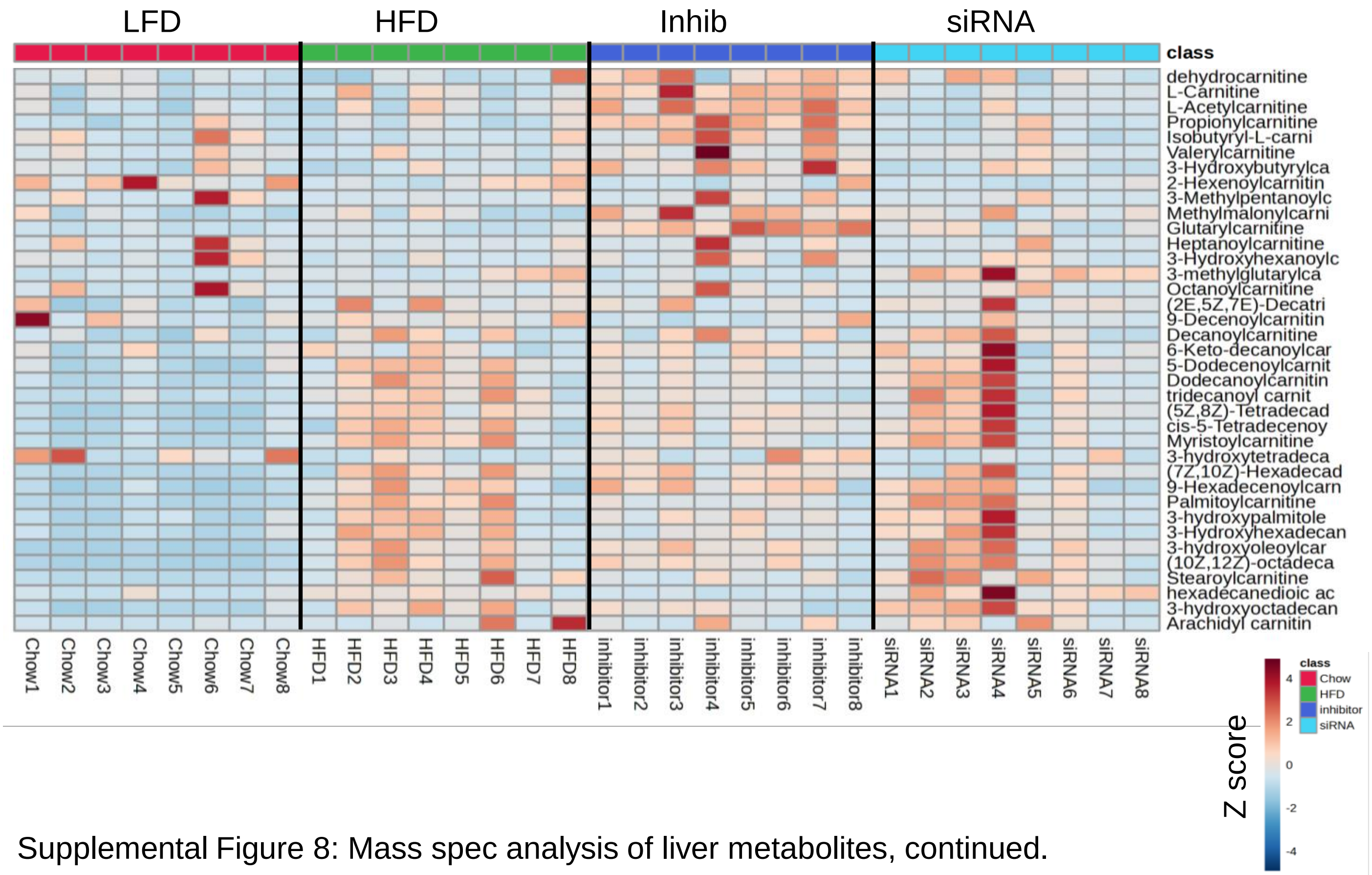

Supplemental Figure 8: Mass spec analysis of liver metabolites, continued.

Heatmap representation of FAO metabolites quantified by mass spectrometry. n = 8 mice per group.

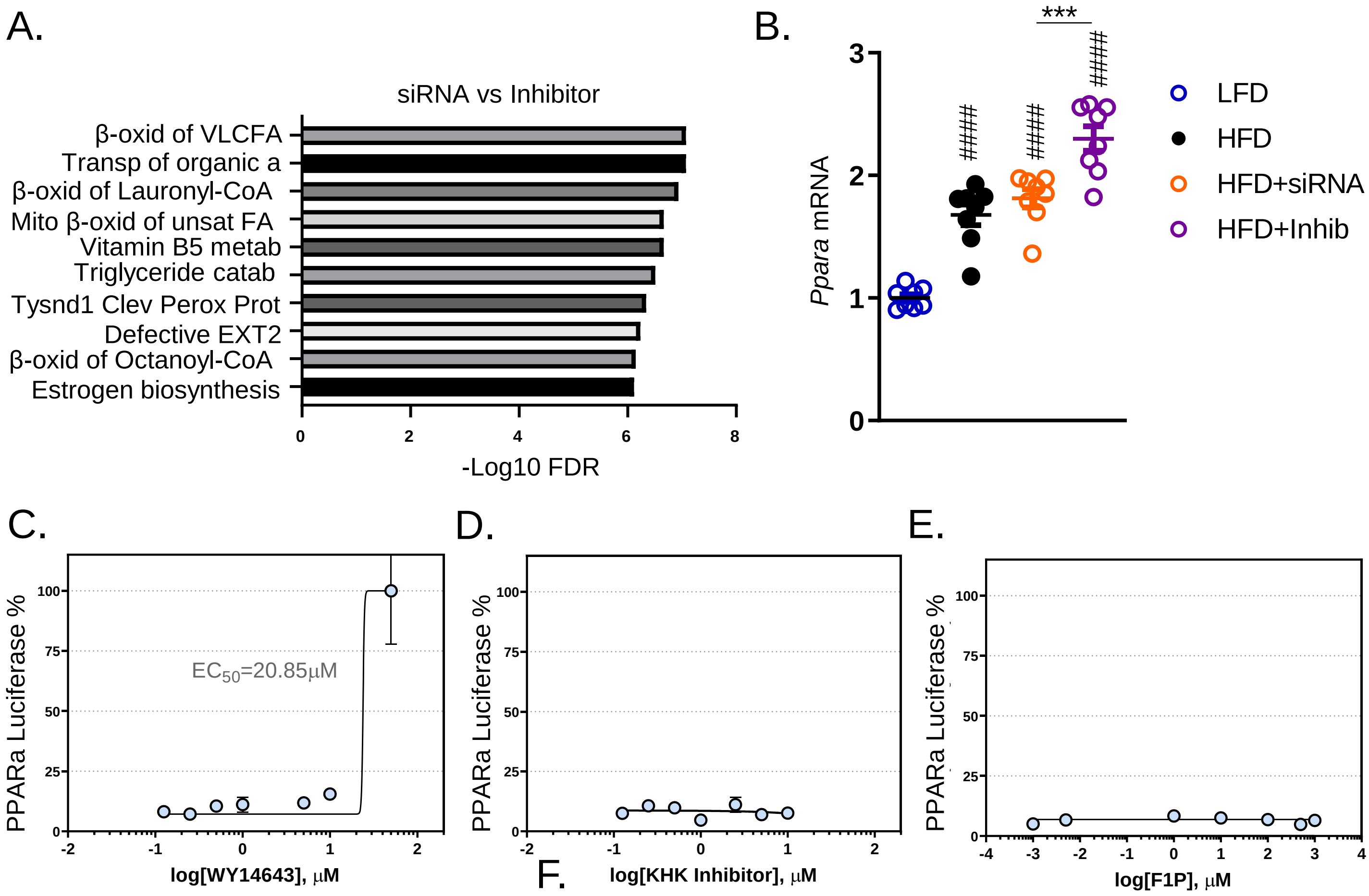

Supplemental Figure 9: PPARa expression and luciferase activity

A) Reactome pathway analysis using RNAseq data comparing siRNA versus the inhibitor treatment. B) Hepatic mRNA expression of PPARa. n = 6 mice per group. The bar graphs show mean ±SEM. # represents statistically significant results by one-way ANOVA compared to the LFD group. * represents post-hock t-test between the groups. C) PPARa luciferase reporter assay following treatment with increasing concentrations of WY14643, D) KHK inhibitor or E) Fructose-1 phosphate (F1P). The highest value of WY14643 was set at 100%. n = 6 reactions per condition.

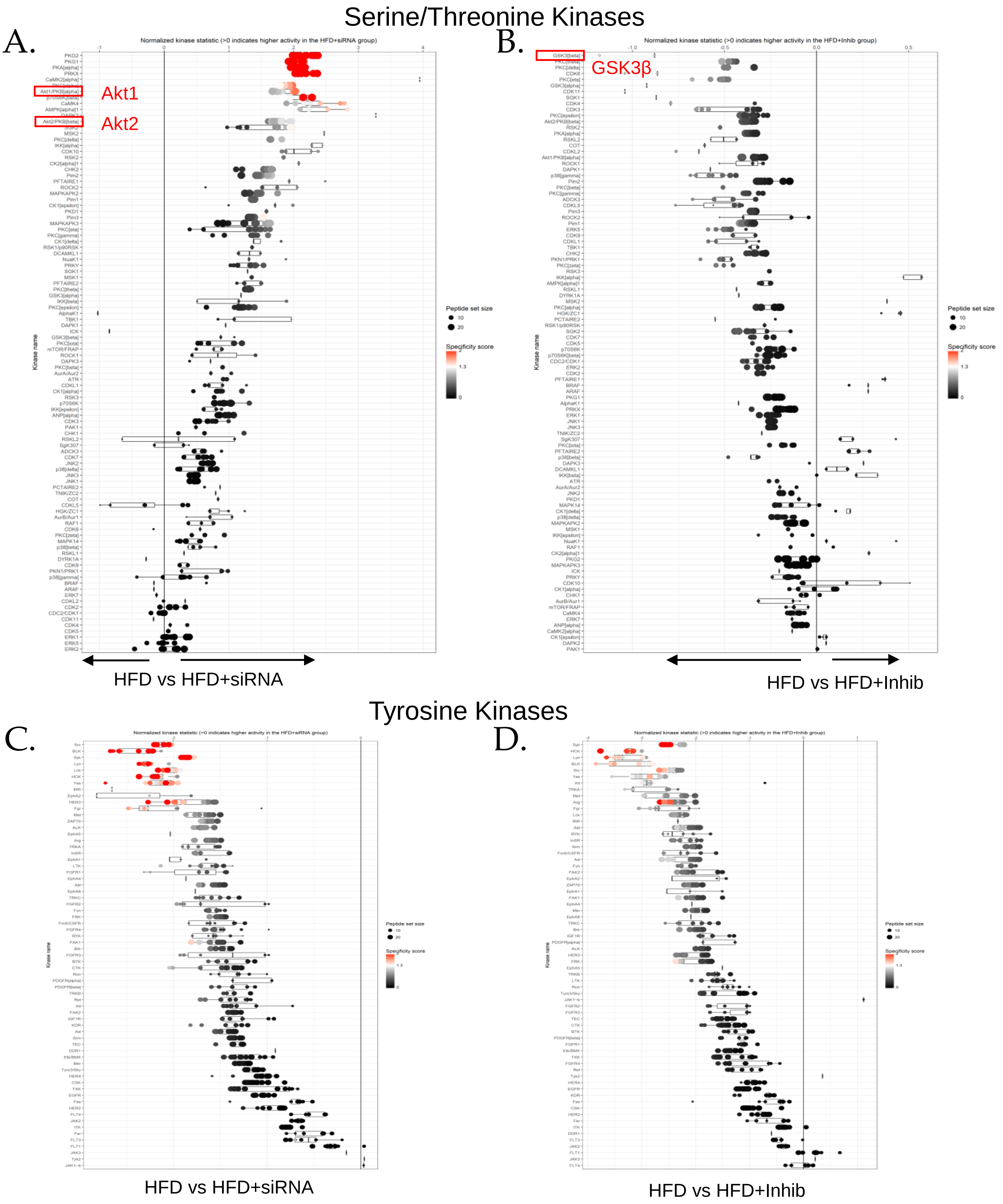

Supplemental Figure 10: Serin/Threonine and tyrosine kinase activities
PamGene PamStation quantification of serine/threonine kinases comparing A) the HFD versus the HFD+siRNA group, as well as B) the HFD versus the HFD+Inhib group. Quantification of tyrosine kinases comparing C) the HFD versus the HFD+siRNA group, as well as D) the HFD versus the HFD+Inhib group. n = 7-8 mice per group.

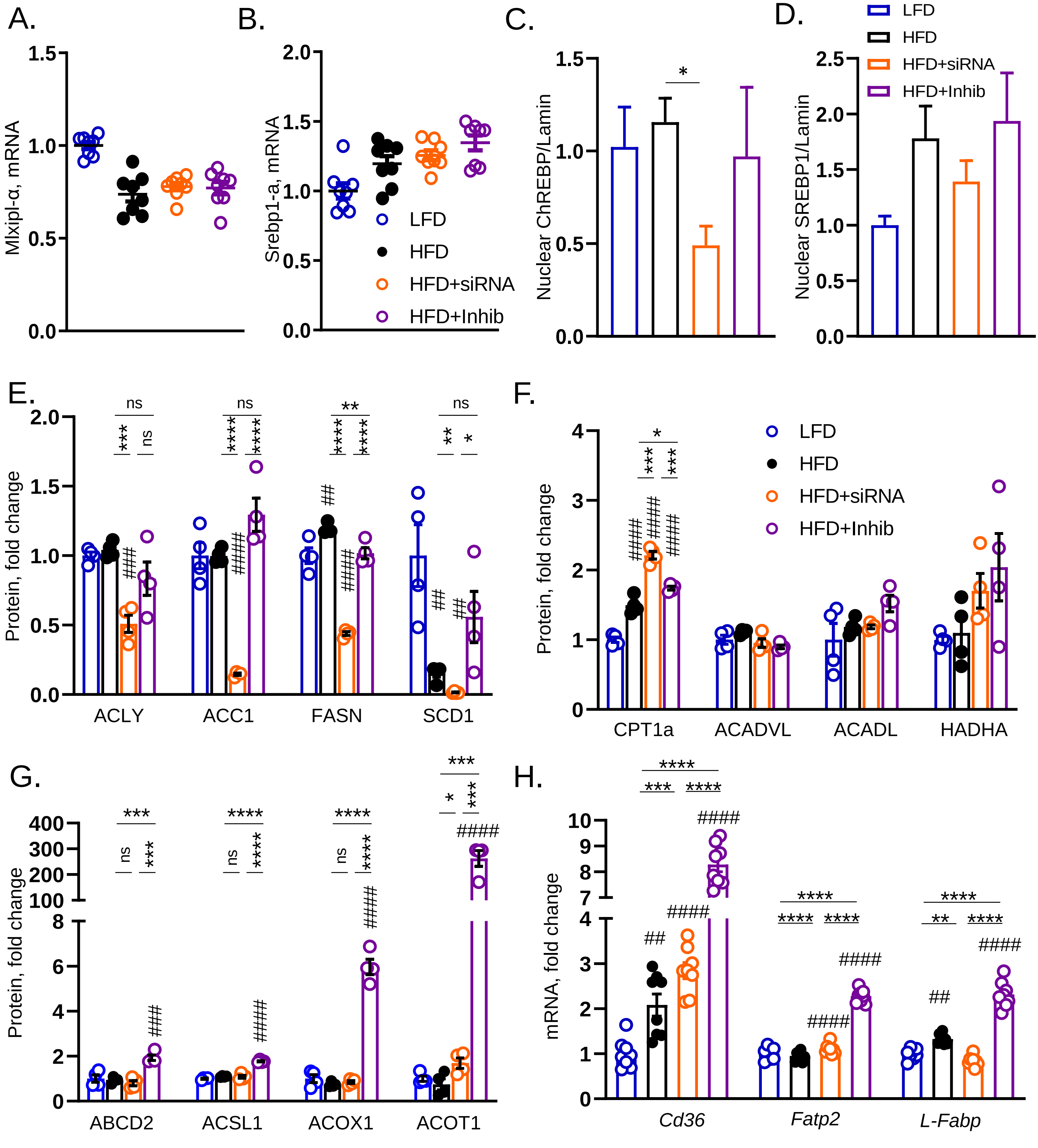

Supplemental Figure 11: Gene expression and protein quantification of the DNL & FAO pathways.

Hepatic mRNA expression of A) Chrebp-a and B) Srebp1a. n = 6 mice per group. C) Densitometry quantification of western blot data using Image J from figure 6C, D) figure 6C, E) figure 6E, F) figure 6F, G) figure H. n = four mice per group. H) Hepatic mRNA expression of the genes regulating fatty acid transport. The bar graphs show mean ±SEM. # represents statistically significant results by one-way ANOVA compared to the LFD group. * represents post-hock t-test between the groups.

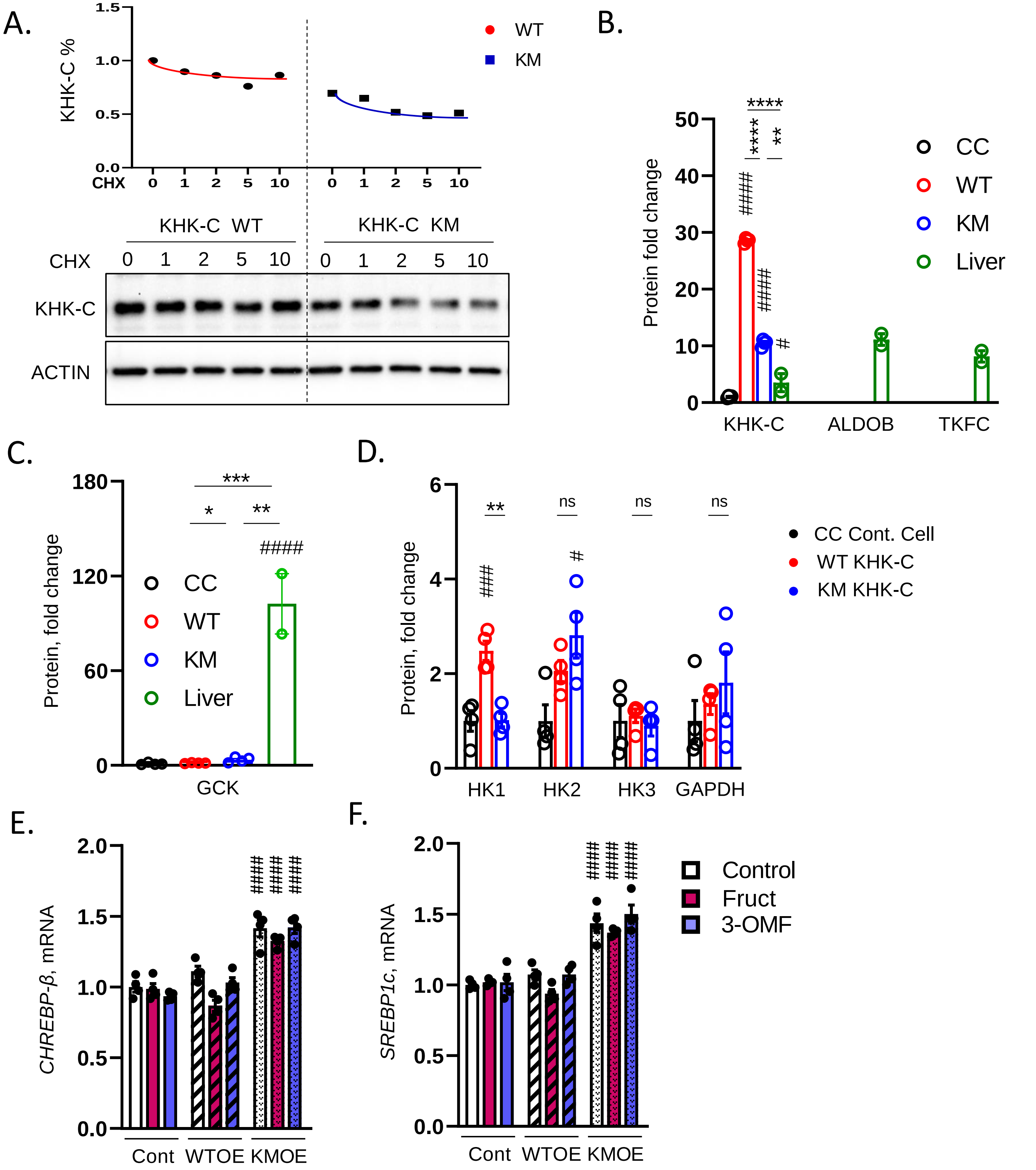

Supplemental Figure 12: Overexpression of WT or KM KHK-C in HepG2 cells.

A) Western blot of KHK-C from wild type (WT) or kinase dead mutant (KM) KHK-C overexpressing cells treated with increasing doses of cycloheximide (CHX). The graph above represents Image J quantification of the data. B) Densitometry quantification of western blot data using Image J from figure 7B, C) figure 7K and D) figure 7K. n = four mice per group. mRNA expression of E) Chrebf and F) Srebp1c. n=4 wells per group. The bar graphs show mean ±SEM. # represents statistically significant results by one way ANOVA compared to the control group. * represents post-hock t-test.
