# Supplementary material for: Knockdown of ketohexokinase versus inhibition of its kinase activity exert divergent effects on fructose metabolism
Source: JCI Insight. 2024 Dec 6;9(23):e184396. doi: 10.1172/jci.insight.184396 (PMC11623947; doi:10.1172/jci.insight.184396)

Figure 2I

T-KHK

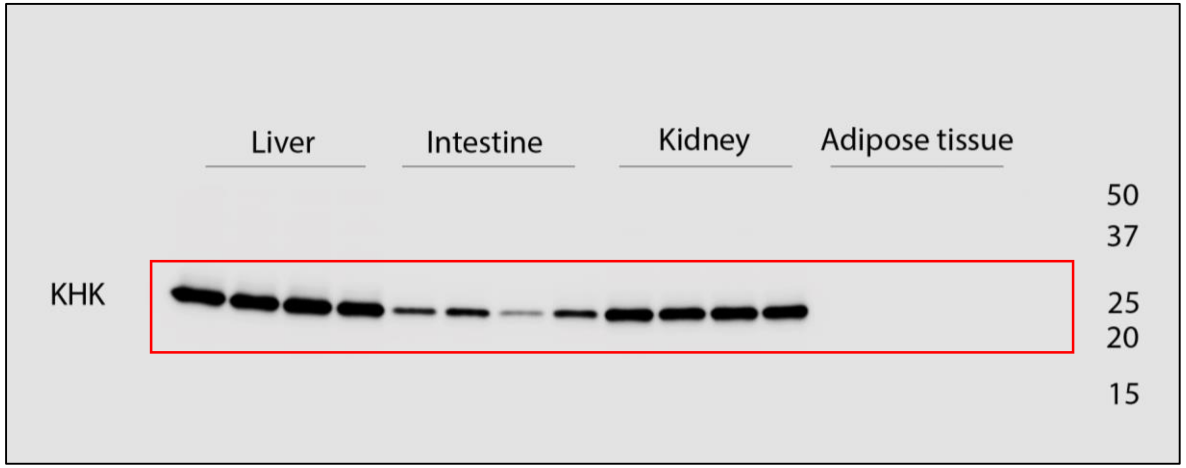

KHK-C

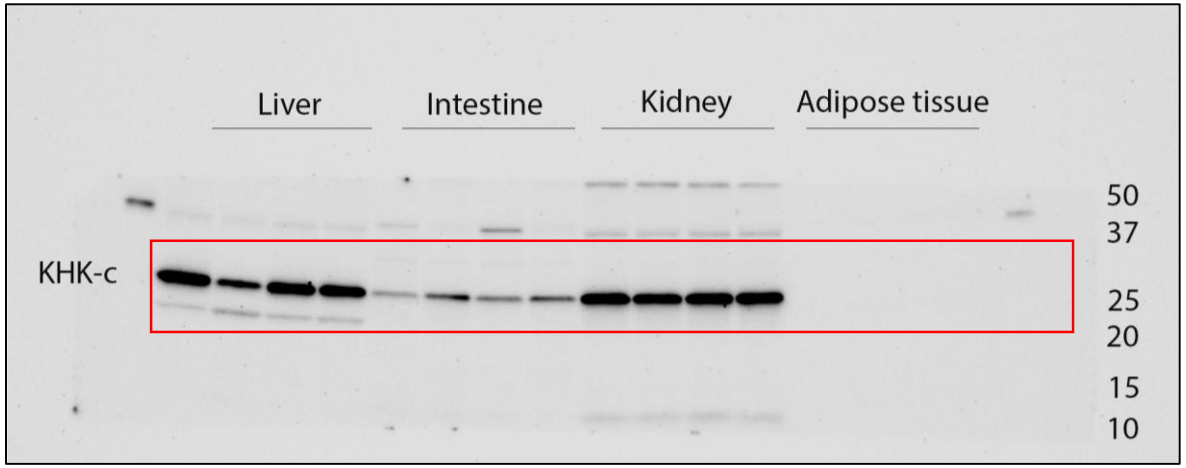

ACTIN

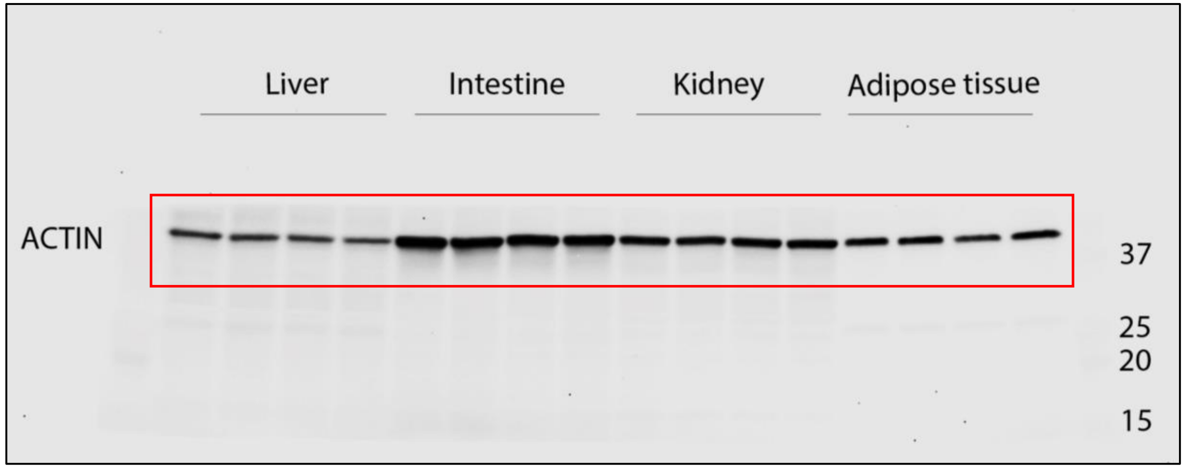

Figure 3C

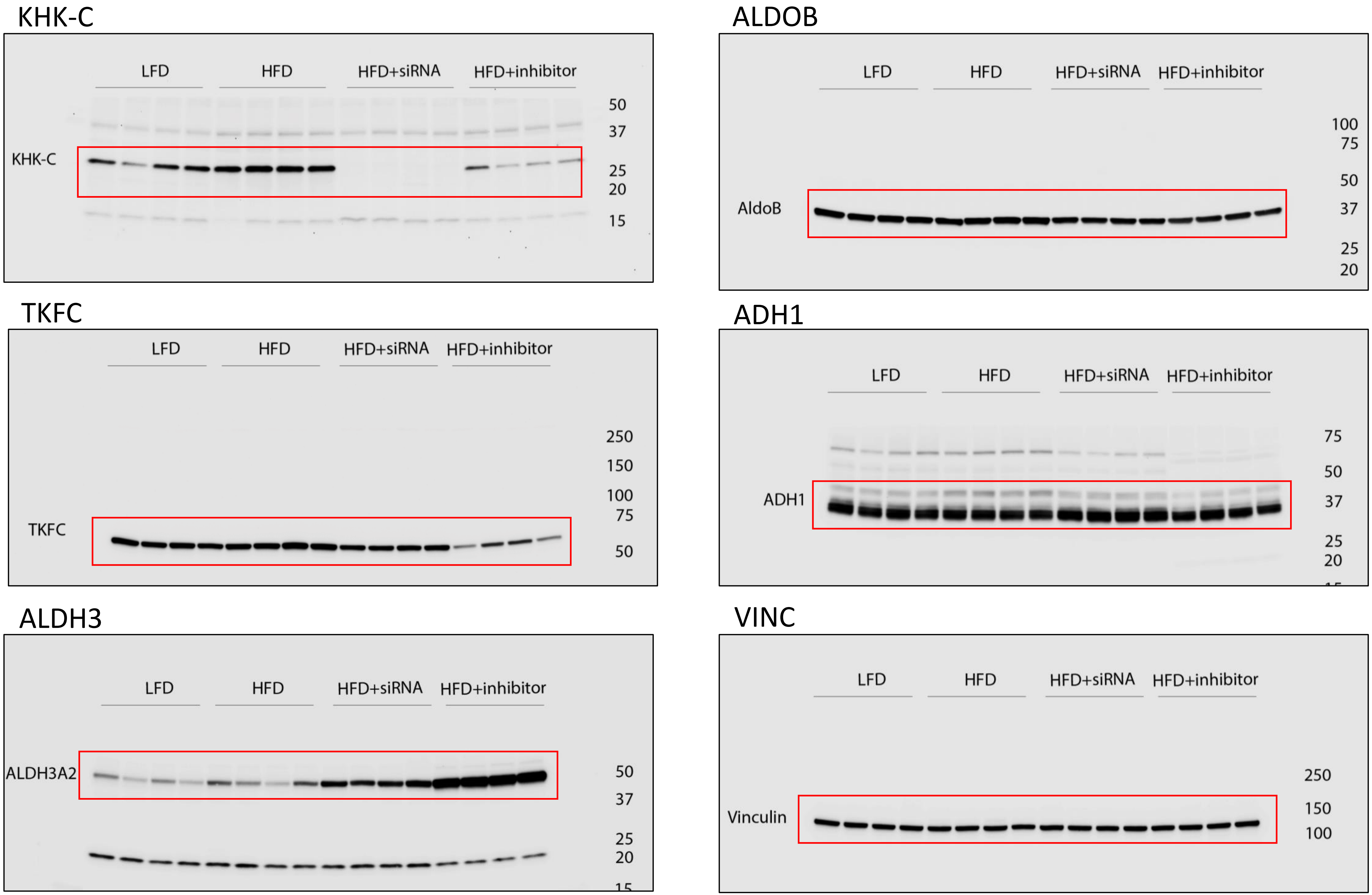
KHK-C
LFD
HFD
HFD+siRNA
HFD+inhibitor
KHK-C
50
37
25
20
15
ALDOB
LFD
HFD
HFD+siRNA
HFD+inhibitor
AldoB
100
75
50
37
25
20
TKFC
LFD
HFD
HFD+siRNA
HFD+inhibitor
TKFC
250
150
100
75
50
ADH1
LFD
HFD
HFD+siRNA
HFD+inhibitor
ADH1
75
50
37
25
20
ALDH3
LFD
HFD
HFD+siRNA
HFD+inhibitor
ALDH3A2
50
37
25
20
VINC
LFD
HFD
HFD+siRNA
HFD+inhibitor
Vinculin
250
150
100

Figure 3F

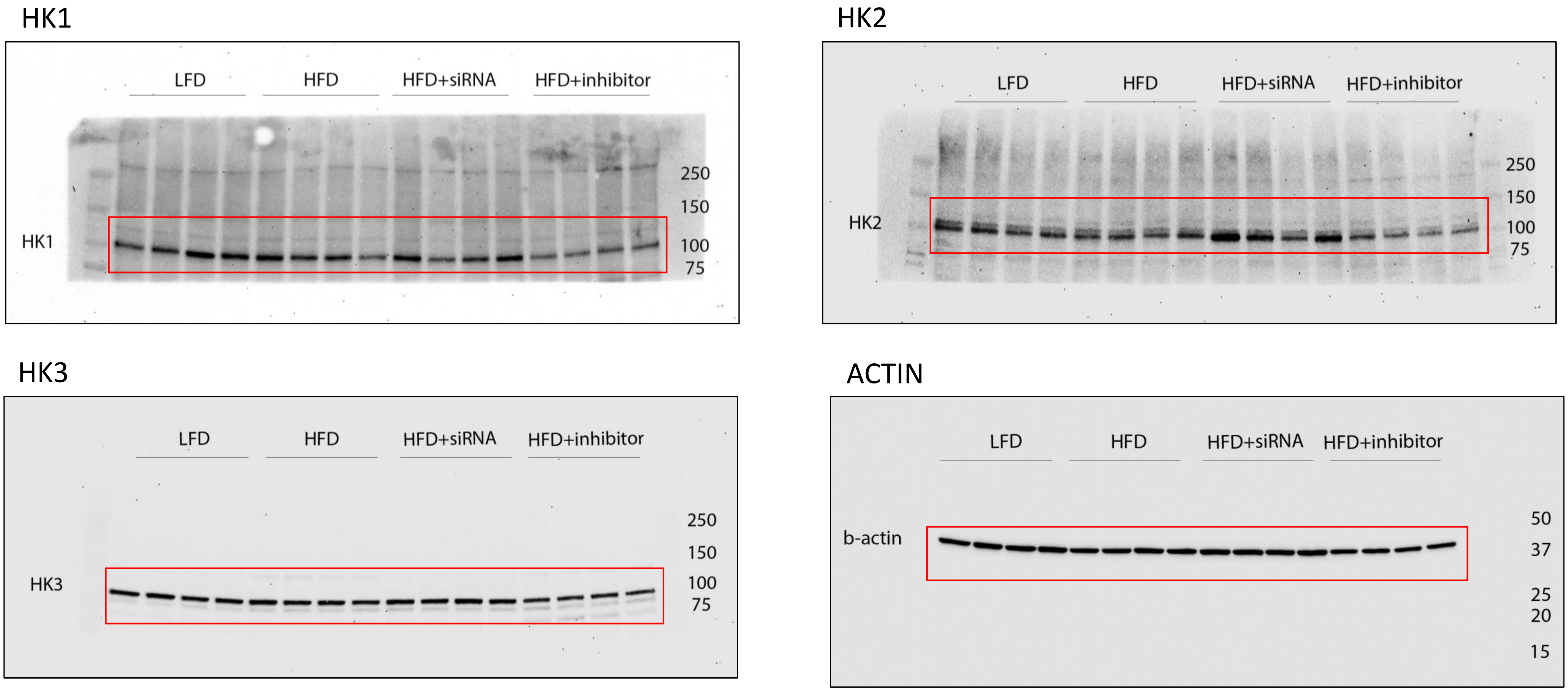
HK1
LFD
HFD
HFD+siRNA
HFD+inhibitor
250
150
HK1
100
75
HK2
LFD
HFD
HFD+siRNA
HFD+inhibitor
250
150
HK2
100
75
HK3
LFD
HFD
HFD+siRNA
HFD+inhibitor
250
150
HK3
100
75
ACTIN
LFD
HFD
HFD+siRNA
HFD+inhibitor
50
b-actin
37
25
20
15

Figure 4F

p-AKT p-ERK1/2 t-AKT t-ERK1/2

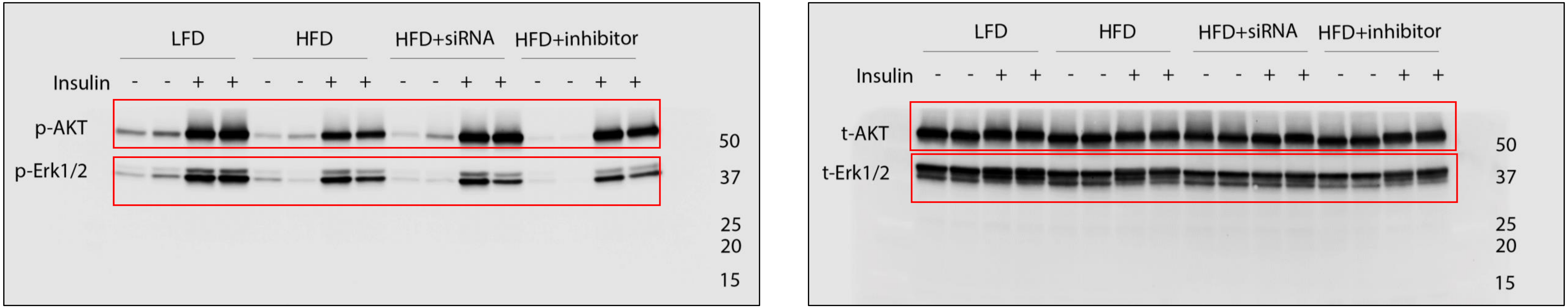

Figure 4G

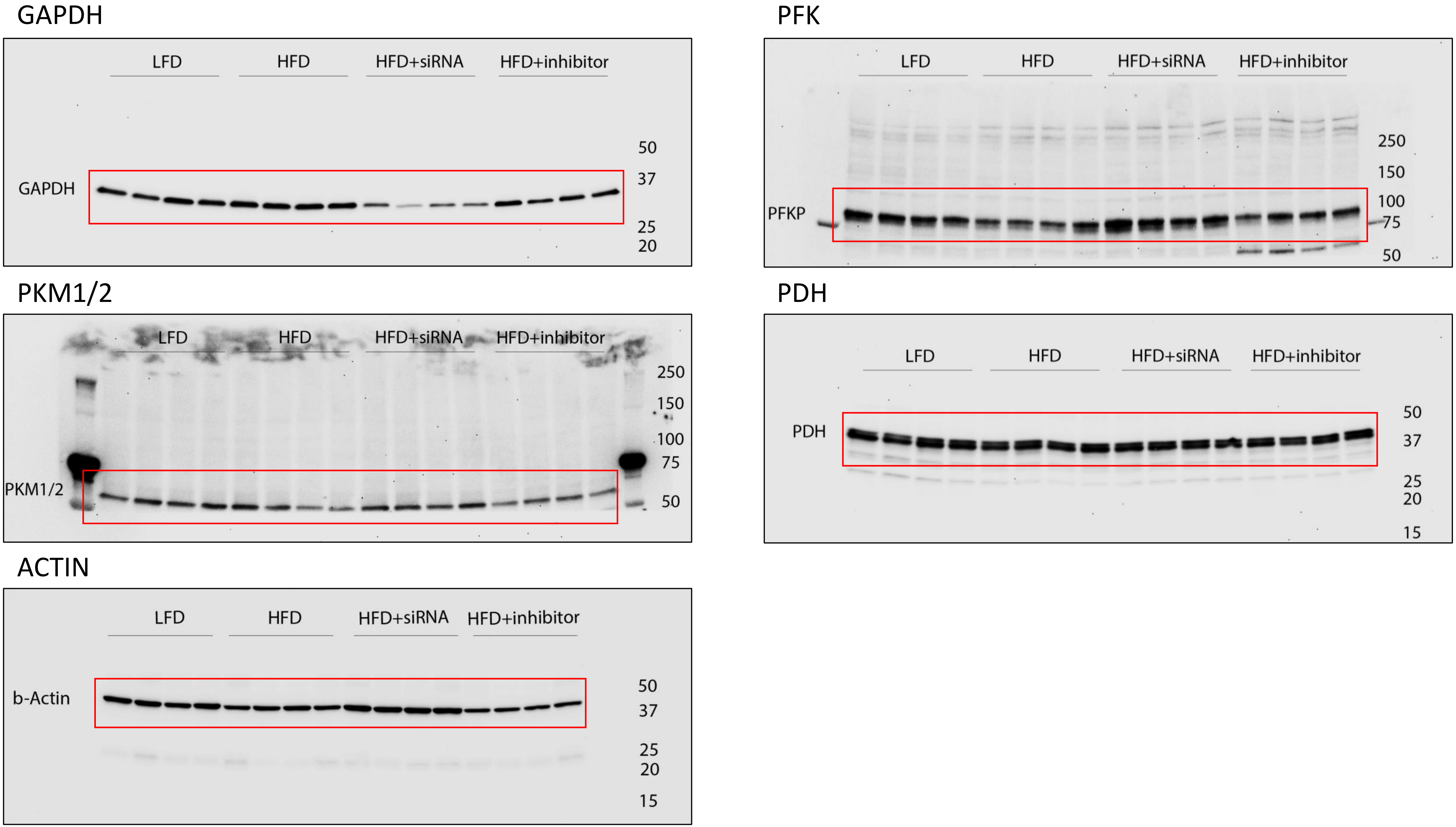

GAPDH
LFD
HFD
HFD+siRNA
HFD+inhibitor
GAPDH
50
37
25
20
PFK
LFD
HFD
HFD+siRNA
HFD+inhibitor
PFKP
250
150
100
75
50
PKM1/2
LFD
HFD
HFD+siRNA
HFD+inhibitor
PKM1/2
250
150
100
75
50
PDH
LFD
HFD
HFD+siRNA
HFD+inhibitor
PDH
50
37
25
20
15
ACTIN
LFD
HFD
HFD+siRNA
HFD+inhibitor
b-Actin
50
37
25
20
15

Figure 6C

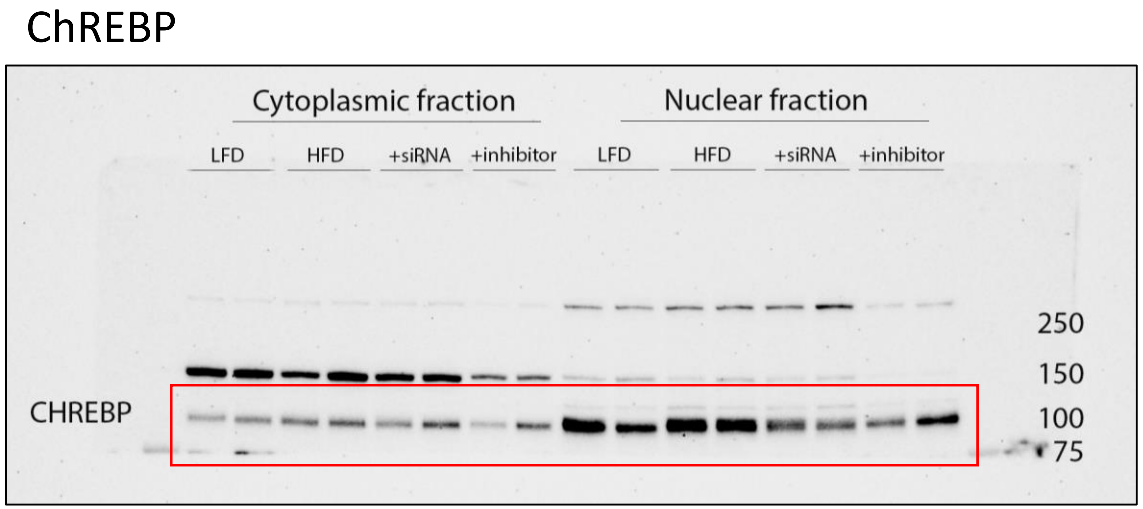
ChREBP
Cytoplasmic fraction
Nuclear fraction
LFD
HFD
+siRNA
+inhibitor
LFD
HFD
+siRNA
+inhibitor
CHREBP
250
150
100
75

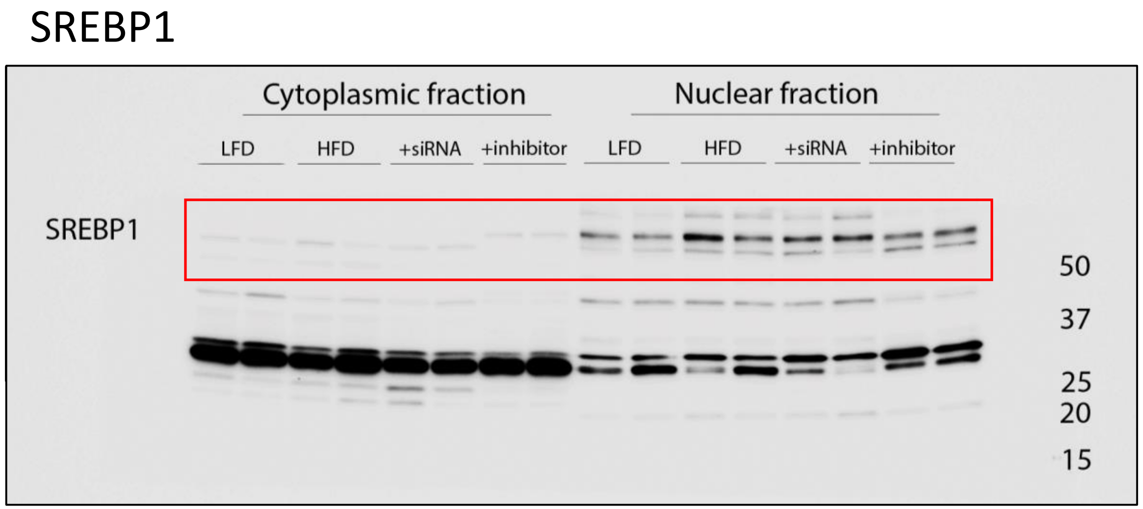
SREBP1
Cytoplasmic fraction
Nuclear fraction
LFD
HFD
+siRNA
+inhibitor
LFD
HFD
+siRNA
+inhibitor
SREBP1
50
37
25
20
15

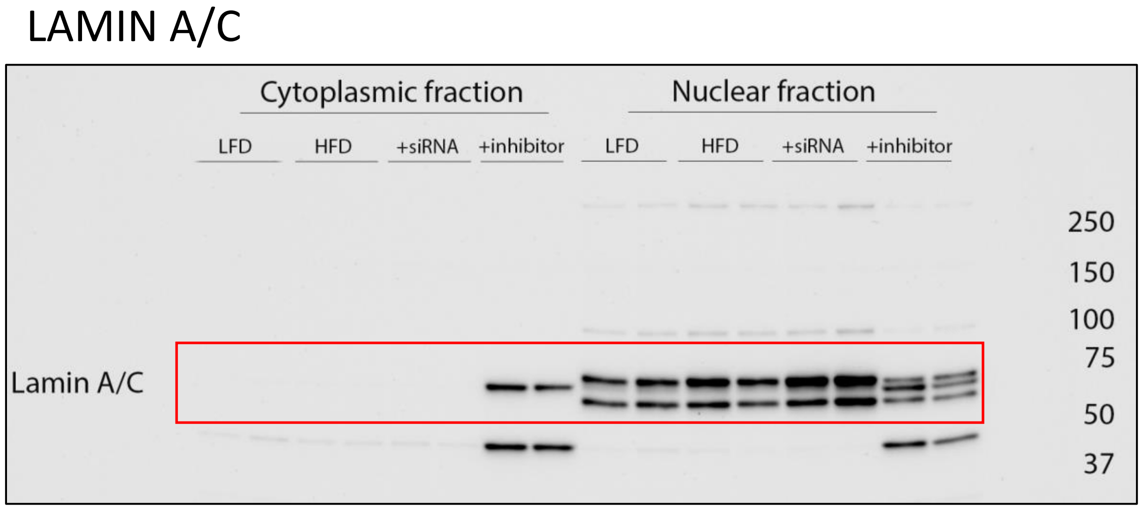
LAMIN A/C
Cytoplasmic fraction
Nuclear fraction
LFD
HFD
+siRNA
+inhibitor
LFD
HFD
+siRNA
+inhibitor
Lamin A/C
250
150
100
75
50
37

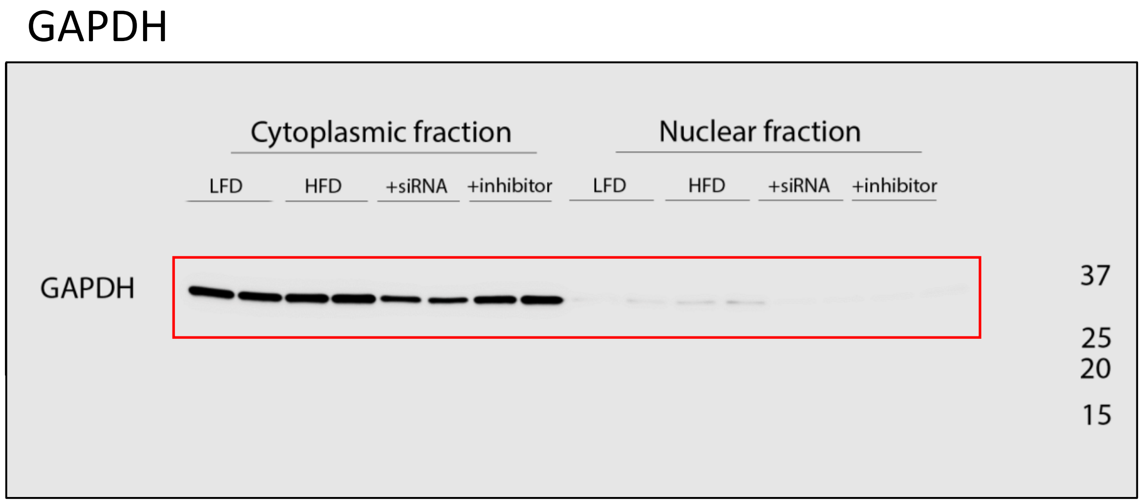
GAPDH
Cytoplasmic fraction
Nuclear fraction
LFD
HFD
+siRNA
+inhibitor
LFD
HFD
+siRNA
+inhibitor
GAPDH
37
25
20
15

Figure 6E

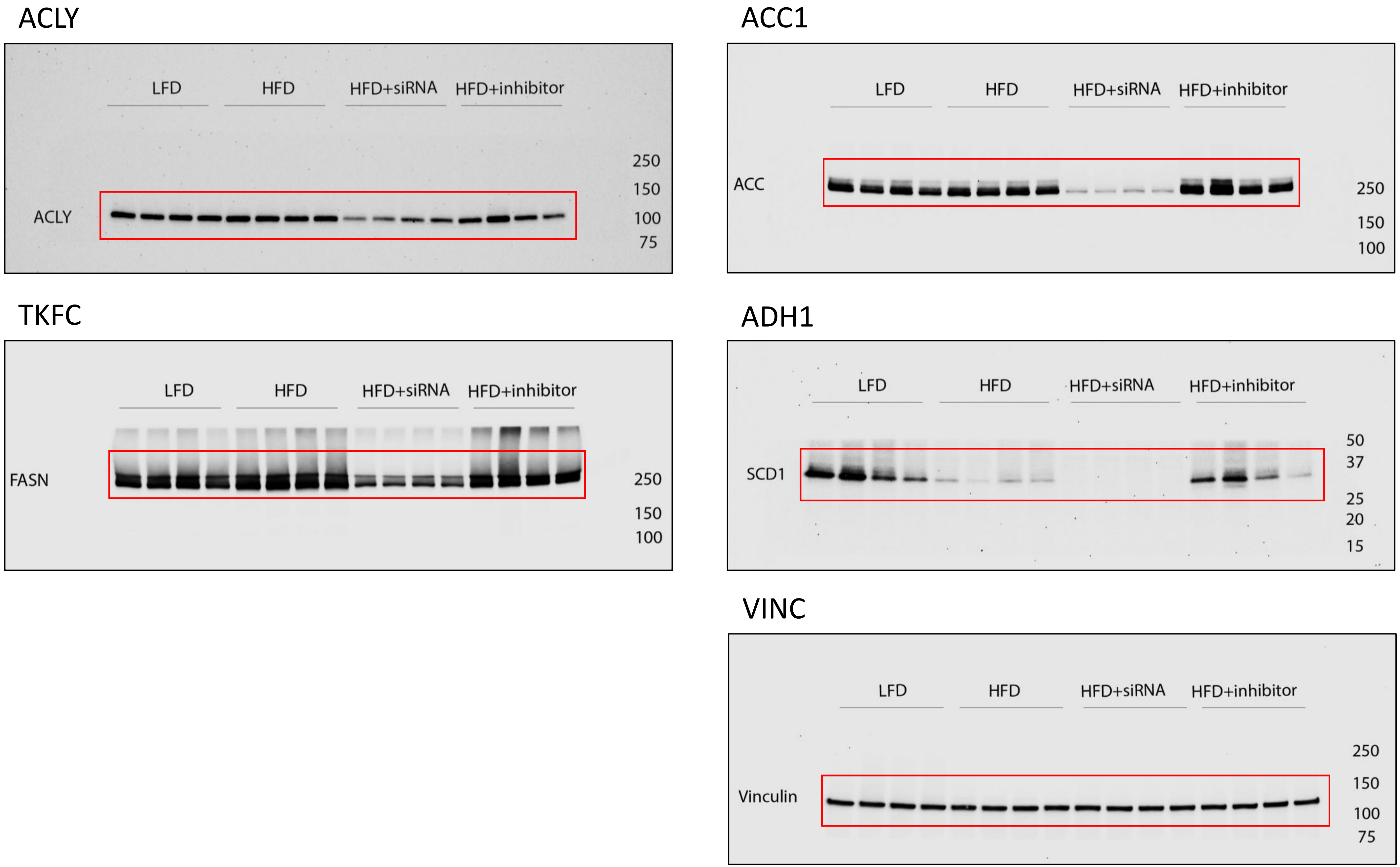

ACLY
LFD
HFD
HFD+siRNA
HFD+inhibitor
ACLY
250
150
100
75
ACC1
LFD
HFD
HFD+siRNA
HFD+inhibitor
ACC
250
150
100
TKFC
LFD
HFD
HFD+siRNA
HFD+inhibitor
FASN
250
150
100
ADH1
LFD
HFD
HFD+siRNA
HFD+inhibitor
SCD1
50
37
25
20
15
VINC
LFD
HFD
HFD+siRNA
HFD+inhibitor
Vinculin
250
150
100
75

Figure 6F

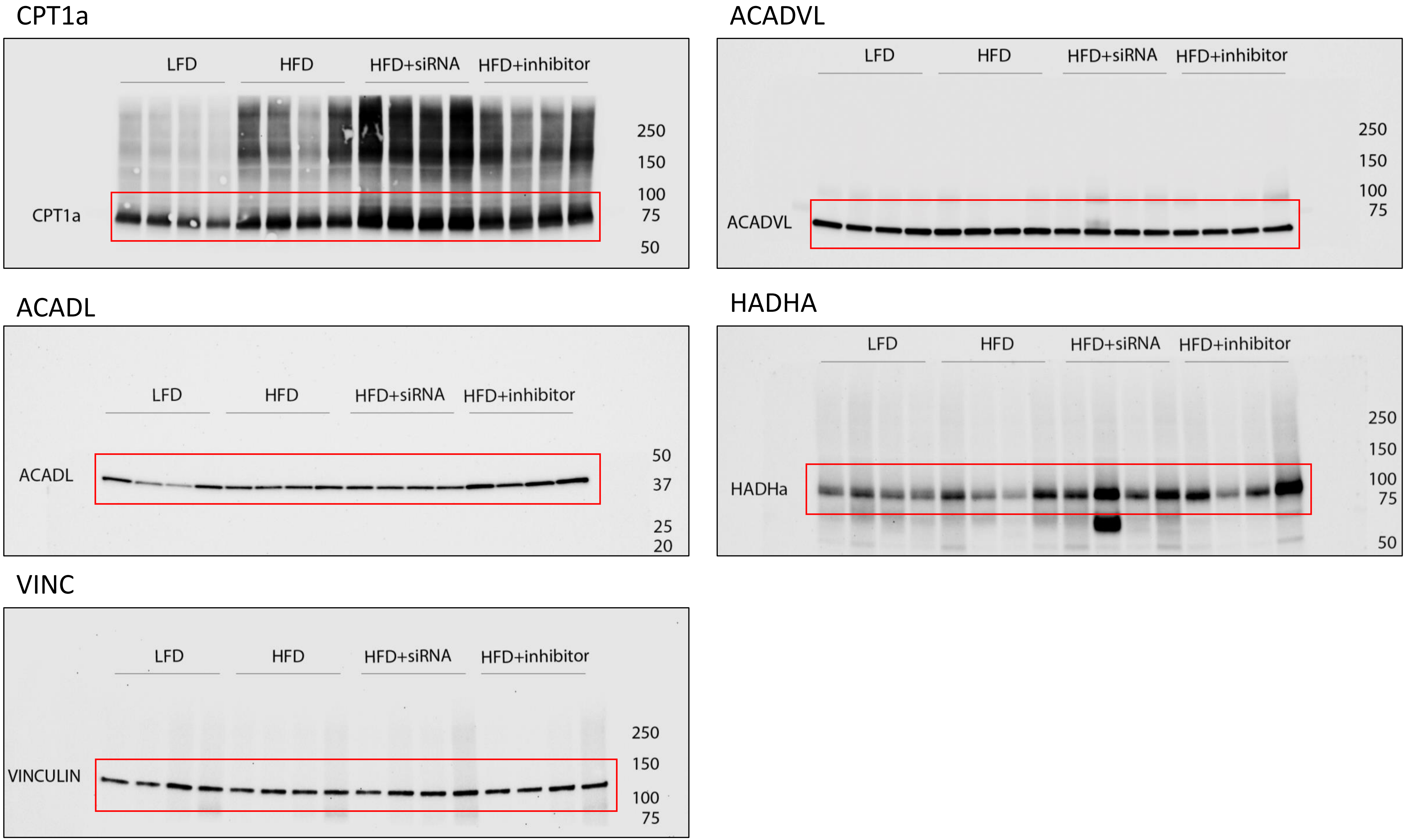
CPT1a
LFD
HFD
HFD+siRNA
HFD+inhibitor
250
150
100
75
50
CPT1a
ACADVL
LFD
HFD
HFD+siRNA
HFD+inhibitor
250
150
100
75
ACADVL
ACADL
LFD
HFD
HFD+siRNA
HFD+inhibitor
50
37
25
20
ACADL
HADHA
LFD
HFD
HFD+siRNA
HFD+inhibitor
250
150
100
75
50
HADHa
VINC
LFD
HFD
HFD+siRNA
HFD+inhibitor
250
150
100
75
VINCULIN

Figure 6H

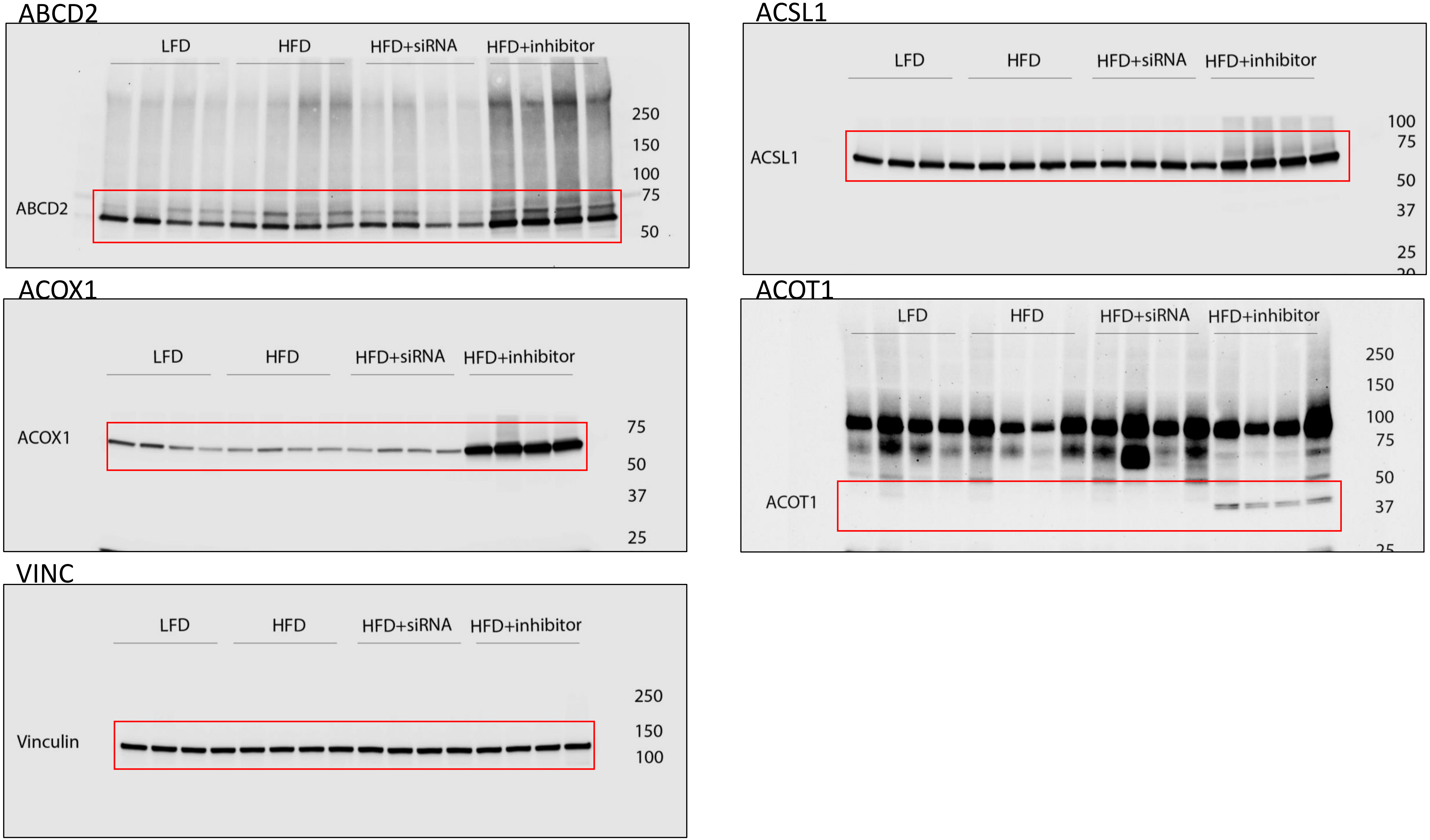
ABCD2
LFD
HFD
HFD+siRNA
HFD+inhibitor
250
150
100
75
50
ABCD2
ACSL1
LFD
HFD
HFD+siRNA
HFD+inhibitor
100
75
50
37
25
ACSL1
ACOX1
LFD
HFD
HFD+siRNA
HFD+inhibitor
ACOX1
75
50
37
25
ACOT1
LFD
HFD
HFD+siRNA
HFD+inhibitor
250
150
100
75
50
37
ACOT1
VINC
LFD
HFD
HFD+siRNA
HFD+inhibitor
250
150
100
Vinculin

Figure 7B

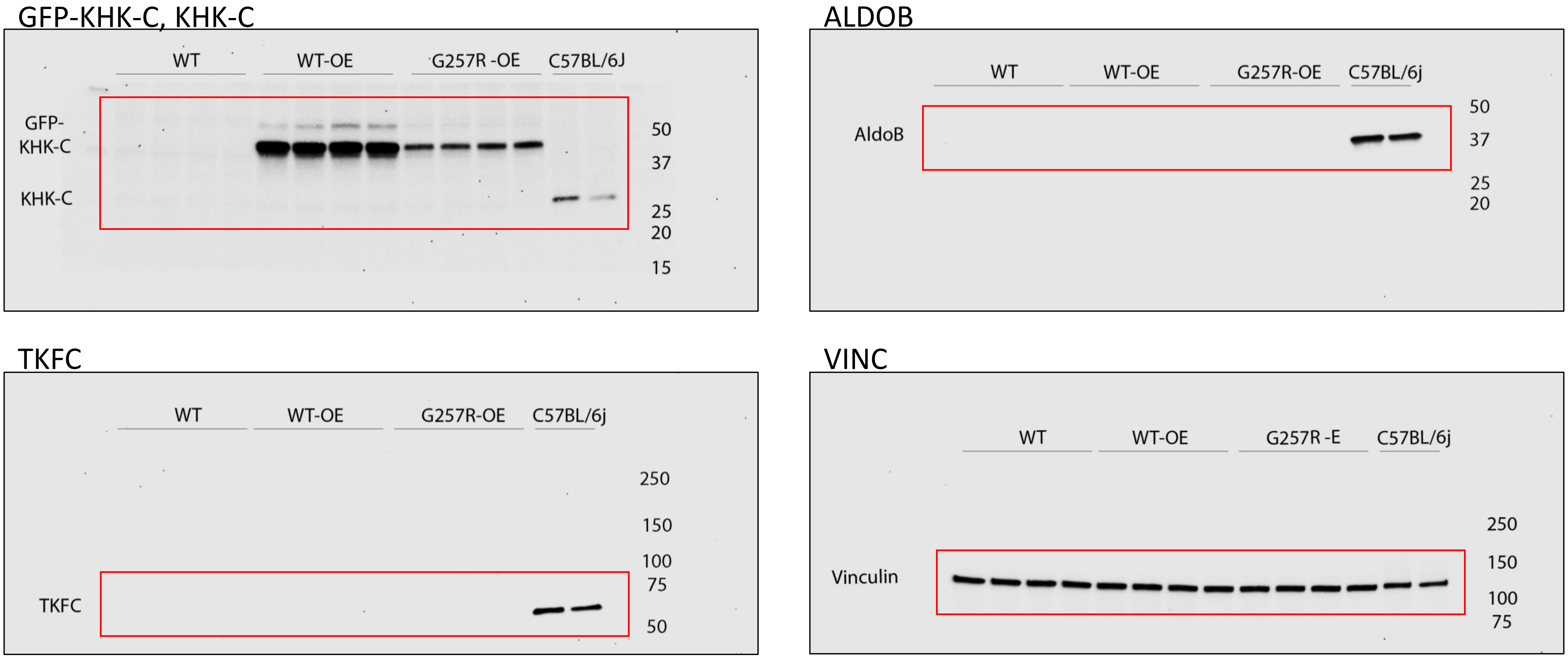
GFP-KHK-C, KHK-C
WT
WT-OE
G257R -OE
C57BL/6J
GFP-
KHK-C
KHK-C
50
37
25
20
15
ALDOB
WT
WT-OE
G257R-OE
C57BL/6j
AldoB
50
37
25
20
TKFC
WT
WT-OE
G257R-OE
C57BL/6j
TKFC
250
150
100
75
50
VINC
WT
WT-OE
G257R -E
C57BL/6j
Vinculin
250
150
100
75

Figure 7K

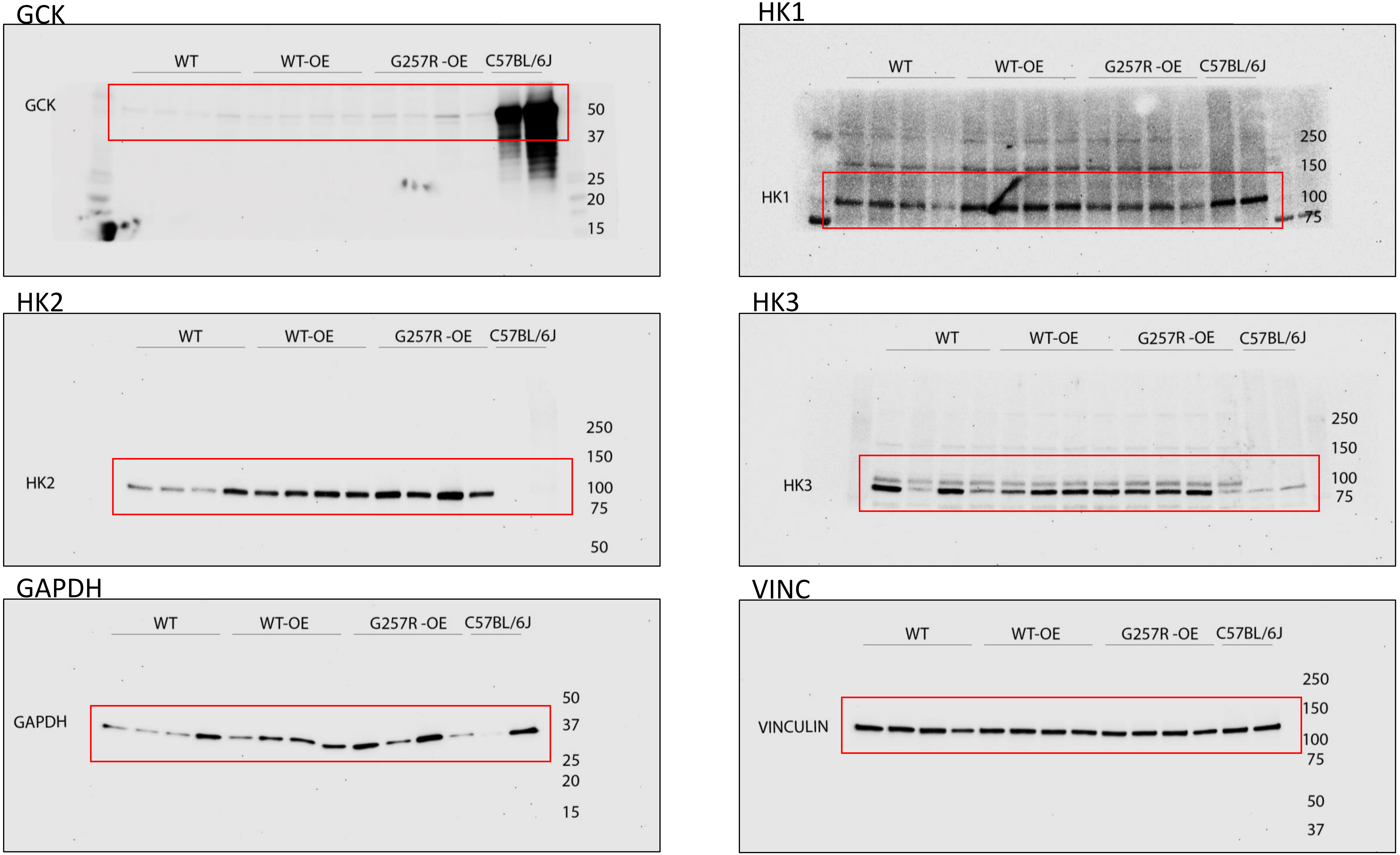
GCK
WT
WT-OE
G257R -OE
C57BL/6J
GCK
50
37
25
20
15
HK1
WT
WT-OE
G257R -OE
C57BL/6J
HK1
250
150
100
75
HK2
WT
WT-OE
G257R -OE
C57BL/6J
HK2
250
150
100
75
50
HK3
WT
WT-OE
G257R -OE
C57BL/6J
HK3
250
150
100
75
GAPDH
WT
WT-OE
G257R -OE
C57BL/6J
GAPDH
50
37
25
20
15
VINC
WT
WT-OE
G257R -OE
C57BL/6J
VINCULIN
250
150
100
75
50
37

Supplemental Figure 4G

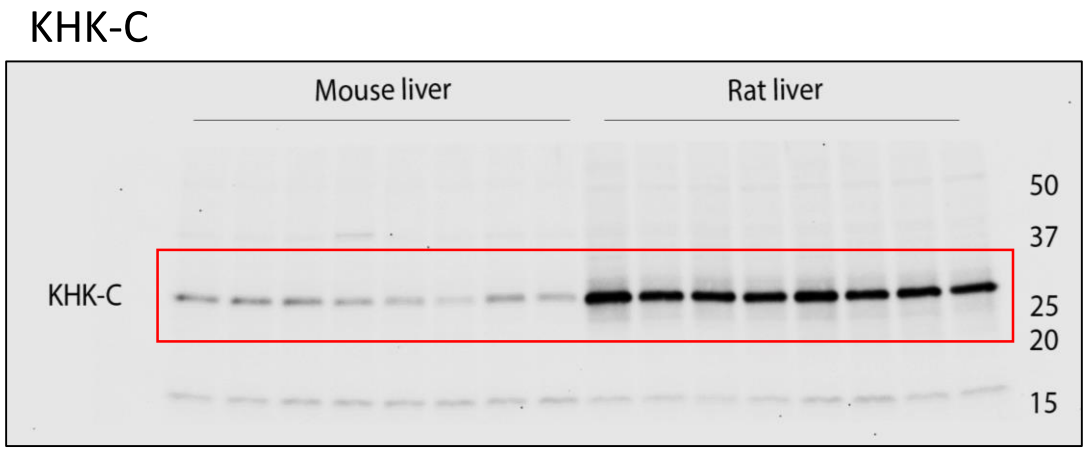

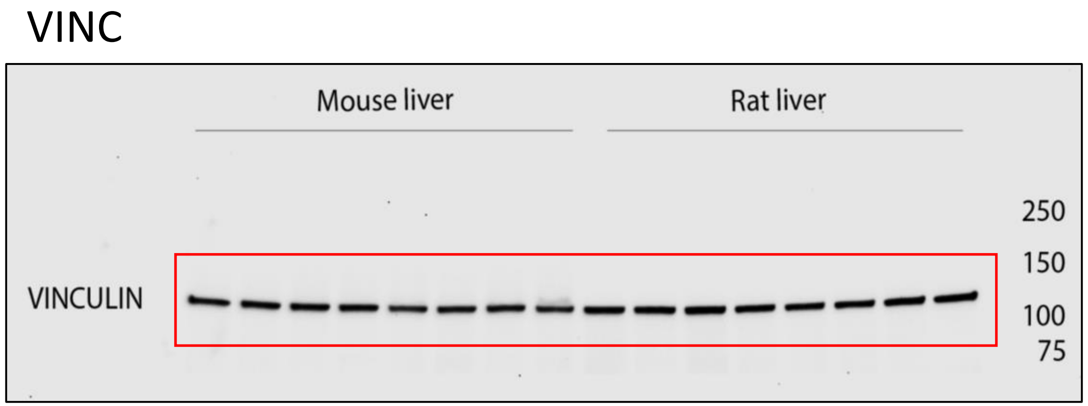

Supplemental Figure 12A

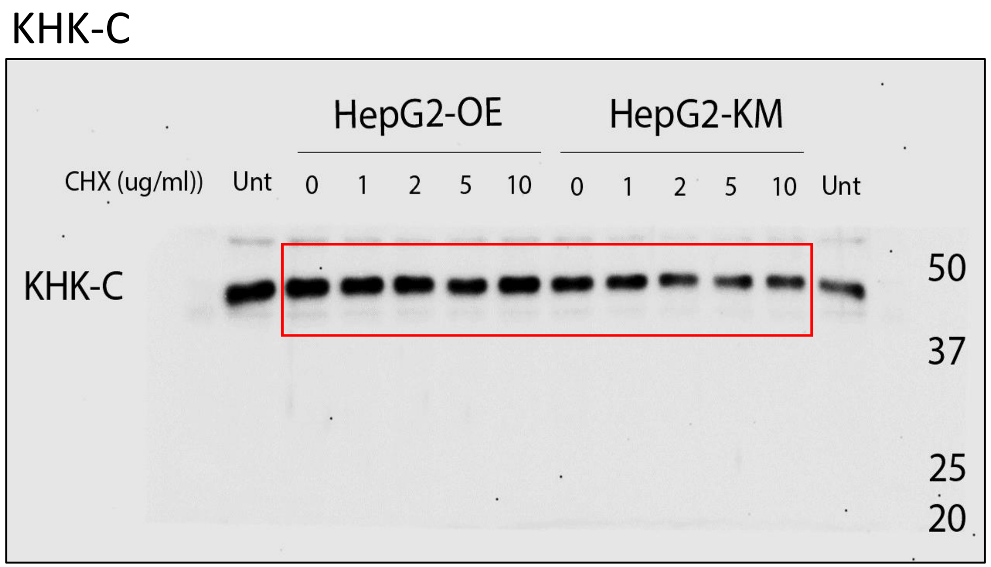

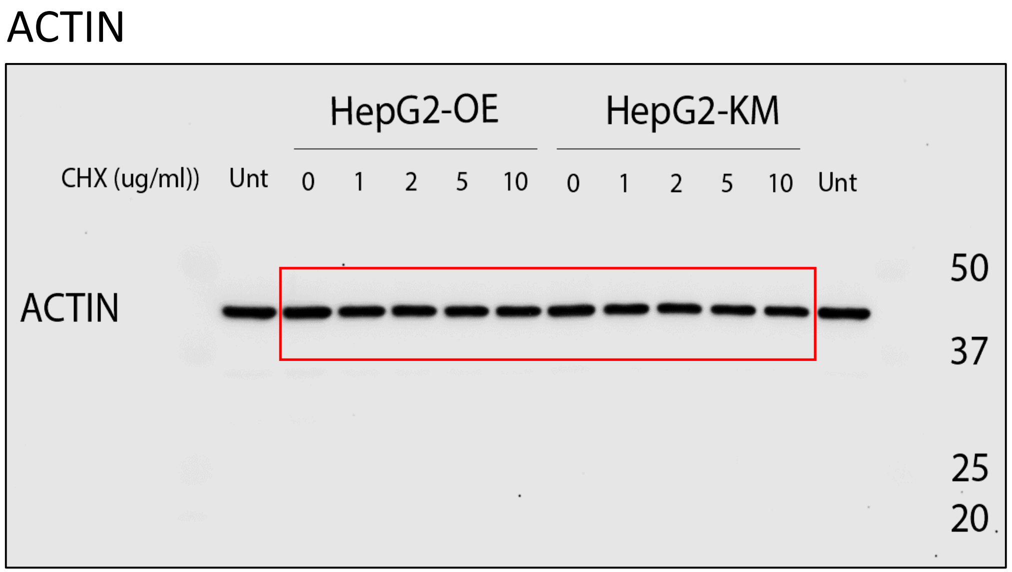

Supplement: Unedited blot and gel images [file jciinsight-9-184396-s171.pdf]
